# Risk factors for human acute leptospirosis in northern Tanzania

**DOI:** 10.1371/journal.pntd.0006372

**Published:** 2018-06-07

**Authors:** Michael J. Maze, Shama Cash-Goldwasser, Matthew P. Rubach, Holly M. Biggs, Renee L. Galloway, Katrina J. Sharples, Kathryn J. Allan, Jo E. B. Halliday, Sarah Cleaveland, Michael C. Shand, Charles Muiruri, Rudovick R. Kazwala, Wilbrod Saganda, Bingileki F. Lwezaula, Blandina T. Mmbaga, Venance P. Maro, John A. Crump

**Affiliations:** 1 Centre for International Health, University of Otago, Dunedin, New Zealand; 2 Kilimanjaro Christian Medical Centre, Moshi, Tanzania; 3 Duke Global Health Institute, Duke University, Durham, North Carolina, United States of America; 4 Division of Infectious Diseases, Duke University Medical Center, Durham, North Carolina, United States of America; 5 Bacterial Special Pathogens Branch, Centers for Disease Control and Prevention, Atlanta, Georgia, United States of America; 6 Department of Mathematics and Statistics, University of Otago, Dunedin, New Zealand; 7 Boyd Orr Centre for Population and Ecosystem Health, Institute of Biodiversity, Animal Health and Comparative Medicine, University of Glasgow, Glasgow, United Kingdom; 8 Department of Veterinary Medicine and Public Health, Sokoine University of Agriculture, Morogoro, Tanzania; 9 Mawenzi Regional Referral Hospital, Moshi, Tanzania; 10 Kilimanjaro Christian Medical University College, Moshi, Tanzania; 11 Kilimanjaro Clinical Research Institute, Moshi, Tanzania; University of California Davis, UNITED STATES

## Abstract

**Introduction:**

Leptospirosis is a major cause of febrile illness in Africa but little is known about risk factors for human infection. We conducted a cross-sectional study to investigate risk factors for acute leptospirosis and *Leptospira* seropositivity among patients with fever attending referral hospitals in northern Tanzania.

**Methods:**

We enrolled patients with fever from two referral hospitals in Moshi, Tanzania, 2012–2014, and performed *Leptospira* microscopic agglutination testing on acute and convalescent serum. Cases of acute leptospirosis were participants with a four-fold rise in antibody titers, or a single reciprocal titer ≥800. Seropositive participants required a single titer ≥100, and controls had titers <100 in both acute and convalescent samples. We administered a questionnaire to assess risk behaviors over the preceding 30 days. We created cumulative scales of exposure to livestock urine, rodents, and surface water, and calculated odds ratios (OR) for individual behaviors and for cumulative exposure variables.

**Results:**

We identified 24 acute cases, 252 seropositive participants, and 592 controls. Rice farming (OR 14.6), cleaning cattle waste (OR 4.3), feeding cattle (OR 3.9), farm work (OR 3.3), and an increasing cattle urine exposure score (OR 1.2 per point) were associated with acute leptospirosis.

**Conclusions:**

In our population, exposure to cattle and rice farming were risk factors for acute leptospirosis. Although further data is needed, these results suggest that cattle may be an important source of human leptospirosis. Further investigation is needed to explore the potential for control of livestock *Leptospira* infection to reduce human disease.

## Author summary

Leptospirosis is an under-recognized but important cause of febrile illness and death in Africa. The bacteria that cause leptospirosis have their usual life cycle in animals; humans are infected as accidental hosts. There is considerable variation between countries as to which reservoir animals and human activities are important for transmission of leptospirosis to humans. In many tropical countries flooding and rodents are the dominant sources of human infection. However, in Africa it is unknown which sources of leptospirosis are most responsible for human infection and what behaviors put people at risk for infection We performed a prospective cross-sectional study, to identify risk factors for acute leptospirosis and sources of human infection. We identified contact with cattle and work in rice fields as risk factors for acute leptospirosis. Our findings indicate that cattle may be an important source for human leptospirosis, and therefore control of leptospirosis in livestock may help prevent leptospirosis in people. Further work is needed to isolate *Leptospira* from humans and livestock. Rice farming was an uncommon activity in our study, but strongly associated with acute leptospirosis. Efforts are warranted to prevent infection in rice farmers living in Africa.

## Introduction

Leptospirosis is a zoonotic bacterial infection and is increasingly recognized as an important cause of fever in Africa [1]. Leptospirosis was a leading cause of severe febrile illness in a study conducted in northern Tanzania during 2007–8, where it was diagnosed in 8.8% of participants [2]. The annual incidence of severe acute leptospirosis in northern Tanzania is high, but has fluctuated during surveillance over two time periods: from 75–102 cases per 100,000 people in 2007–08 to 11–18 cases per 100,000 people in 2012–14, suggesting dynamic transmission patterns [3]. An understanding of major animal reservoirs, sources, and modes of transmission to humans is required to inform leptospirosis control.

Animals infected by *Leptospira* may become carriers and excrete *Leptospira* in urine leading to environmental contamination. Humans can be infected following direct exposure to the urine of infected animals or through contact with contaminated surface water or moist soil [5]. Portals of entry include mucous membranes and broken skin [5]. While the major reservoirs, sources of human infection, and modes of transmission of infection are established on a global scale, there is substantial variation by location reflecting the diverse ecology of *Leptospira*. In many tropical countries, rodent species are considered the most important animal reservoir for human infection [4]. As such, dominant risk factors for leptospirosis in many tropical countries include activities that expose individuals to rodent urine, such as living in urban slums, proximity to sewers, and exposure to flood waters [4, 6, 7]. In Tanzania and most other African countries, the risks factors for human infection are not well characterized [1, 4], and there is some evidence that the risk factors may differ from other tropical countries. In northern Tanzania there is evidence that leptospirosis is more common in rural areas where both livestock and rodents could be important sources of human infection [8], and previous *Leptospira* exposure studies have identified livestock farmers as a high-risk group for *Leptospira* seropositivity [9]. Serogroup reactivity patterns of acute human leptospirosis infections have also suggested that livestock may be reservoirs for human cases [8], and studies of livestock have found high proportions that were seropositive or with leptospiruria [10–12]. To inform leptospirosis control in Tanzania, we aimed to identify risk factors for acute leptospirosis and *Leptospira* seropositivity, and identify sources of human *Leptospira* infection.

## Methods

### Study setting

We conducted a cross-sectional study at Kilimanjaro Christian Medical Centre (KCMC), a 450-bed zonal referral hospital and, Mawenzi Regional Referral Hospital (MRRH) a 300-bed regional referral hospital, both in Moshi. Moshi (population ~180,000) is the administrative capital of the Kilimanjaro Region (population ~1.6 million) of Tanzania. Moshi is situated at approximately 890 meters above sea level and has a tropical climate with rainy seasons from October through December, and March through May. Agriculture in northern Tanzania includes smallholder systems involving mixed crop and livestock farming, as well as pastoralism.

### Study procedures and participants

We enrolled pediatric and adult patients presenting to KCMC and MRRH from February 2012 through May 2014. From Monday through Friday, we screened all patients in the adult medical ward at KCMC and the adult and pediatric medical wards at MRRH within 24 hours of admission, as well as patients presenting to the outpatient department at MRRH. We enrolled consecutive eligible inpatients and every second eligible outpatient. Patients were eligible to participate if they had an axillary temperature of >37.5°C or a tympanic, oral, or rectal temperature of ≥38.0°C at presentation. Inpatients were also eligible if they reported a history of fever within the past 72 hours. After obtaining informed consent, a trained study team member completed standardized clinical history and risk factor questionnaires. The risk factor questionnaire included questions on socio-demographic characteristics, participant living environment, and daily activities performed over the past 30 days, focusing specifically on animal-related activities, exposure to surface water and to rodents (S1 Text). The questionnaire was designed to include established risk factors for leptospirosis from studies done in other settings [4, 6, 7, 13–15], and was piloted prior to use. For participants who lived in the Kilimanjaro Region, study personnel visited participant households to record Global Positioning System (GPS) coordinates of participants’ dwellings. Clinician diagnoses were recorded. Participants were asked to return 4–6 weeks after enrollment for collection of a convalescent serum sample.

### Laboratory methods

Blood was allowed to clot for between 30 and 60 minutes. It was then centrifuged for 15 minutes at 1,126–1455 relative centrifugal force to separate serum. Serum was stored at -80°C. Serum specimens were batch shipped on dry ice from Moshi, Tanzania to Atlanta, GA, United States of America for testing. Serology for leptospirosis was performed at the US Centers for Disease Control and Prevention using the standard microscopic agglutination test (MAT) with a panel of 20 *Leptospira* serovars belonging to 17 serogroups [16]. These included: Australis (represented by *L*. *interrogans* serovar Australis, *L*. *interrogans* serovar Bratislava), Autumnalis (*L*. *interrogans* serovar Autumnalis), Ballum (*L*. *borgpetersenii* serovar Ballum), Bataviae (*L*. *interrogans* serovar Bataviae), Canicola (*L*. *interrogans* serovar Canicola), Celledoni (*L*. *weilii* serovar Celledoni), Cynopteri (*L*. *kirschneri* serovar Cynopteri), Djasiman (*L*. *interrogans* serovar Djasiman), Grippotyphosa (*L*. *interrogans* serovar Grippotyphosa), Hebdomadis (*L*. *santarosai* serovar Borincana), Icterohaemorrhagiae (*L*. *interrogans* serovar Mankarso, *L*. *interrogans* Icterohaemorrhagiae), Javanica (*L*. *borgpetersenii* serovar Javanica), Mini (*L*. *santarosai* serovar Georgia), Pomona (*L*. *interrogans* serovar Pomona), Pyrogenes (*L*. *interrogans* serovar Pyrogenes, *L*. *santarosai* serovar Alexi), Sejroe (*L*. *interrogans* serovar Wolffi), and Tarassovi (*L*. *borgpetersenii* serovar Tarassovi). MAT was performed beginning at a dilution of 1:100, with subsequent two-fold dilutions. Positive and negative controls were included with each run.

### Case definitions

We defined leptospirosis cases as participants with either a four-fold rise in agglutinating antibody titers between acute and convalescent serum, or a single reciprocal titer of ≥800 [17]. Seropositivity was defined as a single positive reciprocal titer of ≥100 from either sample. Controls were participants with negative titers on both acute and convalescent serum samples. The predominant reactive serogroup for cases and seropositive participants was defined as the serogroup containing the serovar with the highest titer.

### Geospatial and rainfall data

For each participant, village population density was calculated from the 2012 Tanzania Population and Housing Census [18]. For the purpose of analysis, *a priori* zone classifications were applied to each village [19]. Villages with a population density of 10 inhabitants/km^2^ were classified as urban; villages ≤15km distance from urban areas with a population density ≥3 and < 10 inhabitants/km^2^ were classified as peri-urban; and villages ≥15km distance from an urban area with a population density of <3 inhabitants/km^2^ [19]. Georeferenced mean annual rainfall and soil type data were obtained from the 2002 Kenya International Livestock Research Institute report [20]. Land use data were obtained from the 2010 National Geomatics Center of China report [21]. Daily rainfall data were obtained from the Tanzania Production Company (TPC) rainfall stations located near Moshi.

### Statistical analysis

Patient history, questionnaire, and MAT data were entered using the Cardiff Teleform system (Cardiff, Inc., Vista, CA, USA) into an Access database (Microsoft Corporation, Redmond, WA, USA). Geospatial data were managed using QGIS, version 2.8.3 (Free Software Foundation, Boston, MA, USA). Spatial scan statistics were calculated using a Bernoulli model to assess evidence of spatial clustering of cases using SatScan version 9.0 (www.satscan.org) [22]. All other analyses were performed using Stata, version 13.1 (StataCorp, College Station, TX, USA).

#### Modeling strategy

Logistic regression was used to investigate associations between independent variables and two separate outcome measures: acute leptospirosis and *Leptospira* seropositivity. Initially all associations between individual behavior variables and our outcome variables were assessed by bivariable logistic regression. In addition, to understand the relationship between independent variables, we performed bivariable logistic regression between all independent variables. We then developed models to investigate the behavioral variables and the geospatial variables separately. Because of the high ratio of independent variables to cases of acute leptospirosis, we considered multivariable logistic regression models of the individual behavior variables, and the georeferenced variables to be unstable. Therefore, to facilitate multivariable modeling, and to assess the effects of cumulative exposure we created summary scales to quantitatively estimate overall patient exposure to each of the main modes and sources of infection that we identified from the published literature: urine of cattle, goats, pigs and rodents, and surface water [4]. This meant that when analyzing risk factors for acute leptospirosis we built a single multivariable model using the exposure scores that are described in detail below. When analyzing risk factors for *Leptospira* seropositivity, we were able to build three multivariable models: one multivariable model using individual behavioral risk factors, one model using individual geospatial risk factors, and one model using the exposure scores.

#### Development of exposure scores

We used an analytic hierarchy process to develop these scales [23]. First we identified relevant behaviors and living conditions from the risk factor questionnaire to be included in each scale. We then identified locally experienced subject matter experts, including livestock field officers, physicians, rodent ecologists, veterinarians, water engineers, water and sanitation epidemiologists, and zoonotic disease epidemiologists. For each potential source of infection we asked experts to rank each relevant behavior against every other behavior in terms of the likelihood and intensity of exposure to the source using a 9-point bidirectional scale. We then calculated weightings using a matrix (Appendices 2–4) that added, for each behavior, reciprocals of the score from each pairwise comparison [24]. We assessed precision and error in the judgment process through measuring an expert’s internal consistency in multiple pairwise comparisons. We excluded weightings by experts who provided internally inconsistent answers, as designated by a consistency ratio >0.2 [24], and then calculated the geometric mean of the weightings given by all included experts. To aid interpretation of exposure scores, we multiplied all weights within each scale by a constant, so that possible scores on each scale ranged from 0 to 5. Finally, we derived an overall score for each participant, on each exposure scale, based on their questionnaire answers, such that someone who had performed none of the exposure activities scored zero, and someone who performed all of the activities scored 5.

#### Model building using exposure scales

Relationships between exposure scores and log odds of acute leptospirosis and *Leptospira* seropositivity, were assessed using fractional polynomial transformations of the exposure scales [25]. We allowed up to two degrees of freedom, and utilized the function selection procedure of Stata’s multivariable fractional polynomial algorithm to select the best fitting transformation for each exposure scale. The correlation between exposure scales was assessed using linear regression. Interactions between exposure scales were assessed using factor variables. Through use of directed acyclic graphs, we considered that all exposure scores might act as confounders, and our initial multivariable models included all exposure scales. Variables were examined for co-linearity using variance inflation factors. We used stepwise backwards elimination to arrive at the model that minimized the Akaike Information Criterion (AIC) [26], p values were two sided, and the significance level was set at 0.05.

#### Model building using individual behaviors, and geospatial risk factors

Our initial models of individual behavioral and our initial models of geospatial risk factors included all variables with a p value <0.2 in bivariable logistic regression. No variables were forced into the model as confounders. We performed backwards stepwise model selection to minimize the AIC.

### Research ethics

This study was conducted in accordance with the Declaration of Helsinki. It was approved by the KCMC Research Ethics Committee (#295), the Tanzania National Institute for Medical Research National Ethics Coordinating Committee (NIMR1HQ/R.8cNo1. 11/283), Duke University Medical Center Institutional Review Board (IRB#Pro00016134), and the University of Otago Human Ethics Committee (Health) (H15/055). Written informed consent was obtained from all participants or their guardians.

## Results

### Enrollment and participant characteristics

**Fig 1:**
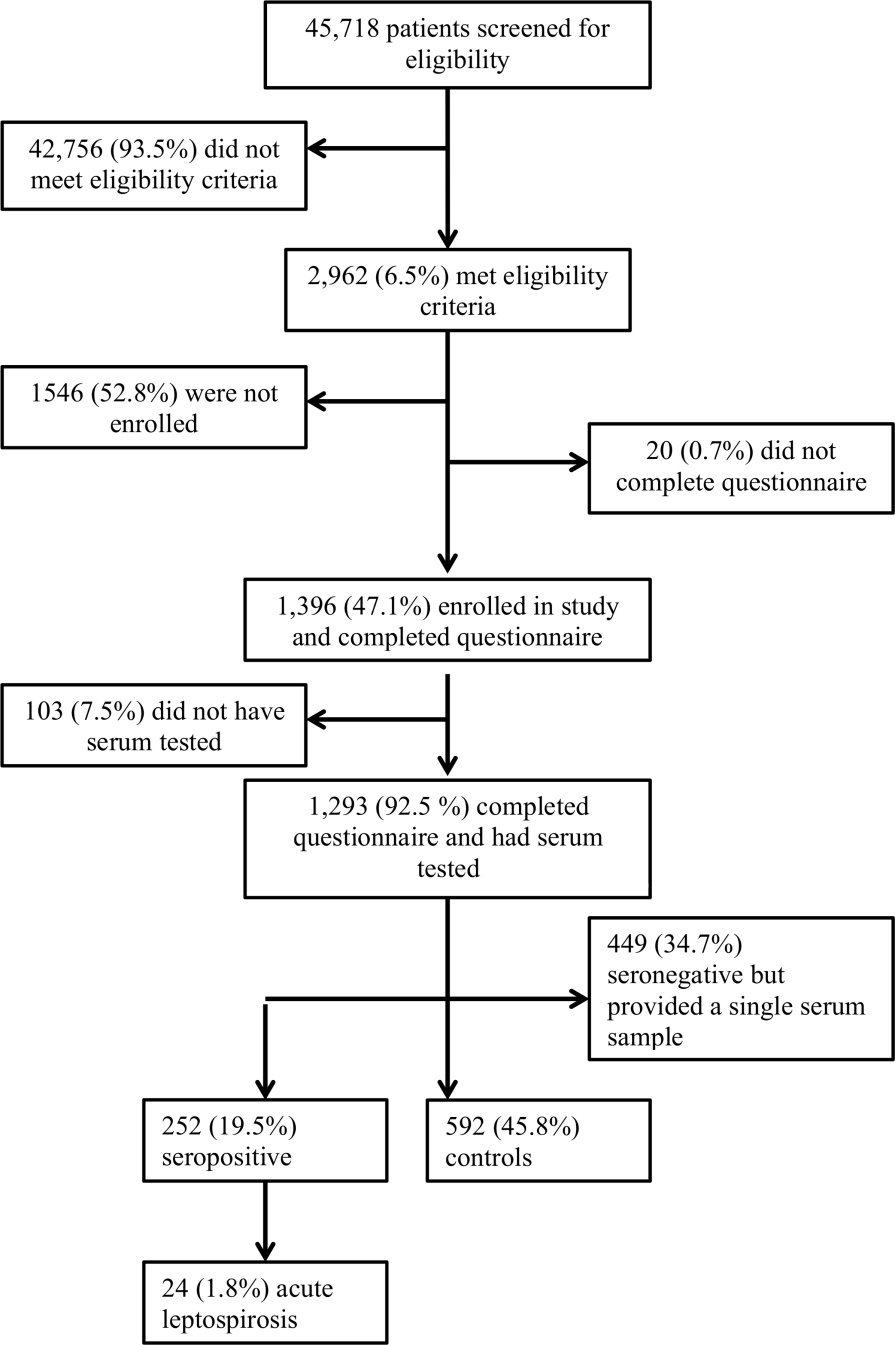
Study flow diagram for patients seeking care at Kilimanjaro Christian Medical Centre and Mawenzi Regional referral hospital in Moshi, Tanzania, 2012–14.

Of 15,305 patients admitted and 30,413 presenting to the outpatient department, 2,962 met eligibility criteria and 1,416 (47.8%) were enrolled. Of 1,293 participants who completed the risk factor questionnaire and had serum tested, 24 (1.9%) met the study criteria for acute leptospirosis, 252 (19.5%) were seropositive, and 592 (45.8%) were classified as controls (Fig 1). The remaining 449 (34.7%) were seronegative but provided only a single serum sample and so were excluded from analysis. The frequency with which participants were predominantly reactive to different serogroups is shown in Table 1. Participant characteristics are shown in Table 2. Clinicians did not diagnose leptospirosis in any study participant. Four (25.0%) of 16 leptospirosis cases with discharge diagnoses recorded were diagnosed with malaria despite negative blood parasite examinations.

**Table 1:** Predominantly reactive serogroup of leptospirosis cases and participants seropositive to *Leptospira*, northern Tanzania, 2012–14.

Serogroup	Leptospira cases (N = 24)			*Leptospira* seropositive (N = 271)*		
	n	(%)	(95% CI)	N	(%)	(95% CI)
Australis	9	(37.5)	(19.8–59.4)	66	(24.4)	(19.6–29.9)
Sejroe	4	(16.7)	(5.9–38.9)	11	(4.1)	(2.3–7.2)
Icterohaemorrhagiae	3	(12.5)	(3.7–34.5)	151	(55.7)	(49.7–61.6)
Djasiman	3	(12.5)	(3.7–34.5)	13	(4.8)	(2.8–8.1)
Pyrogenes	2	(8.3)	(1.9–30.2)	4	(1.5)	(0.6–3.9)
Grippotyphosa	2	(8.3)	(1.9–30.2)	2	(0.7)	(0.2–2.9)
Tarassovi	1	(4.2)	(0.5–27.4)	4	(1.5)	(0.6–3.9)
Mini	0	(0)	-	7	(2.6)	(1.2–5.3)
Bataviae	0	(0)	-	4	(1.5)	(0.6–3.9)
Canicola	0	(0)	-	3	(1.1)	(0.4–3.4)
Autumnalis	0	(0)	-	3	(1.1)	(0.4–3.4)
Hebdomadis	0	(0)	-	2	(0.7)	(0.2–2.9)
Celledoni	0	(0)	-	1	(0.4)	(0.1–2.6)

*Of 252 seropositive participants, 17 individuals had equal titers to 2 serogroups, and 1 individual had equal titers to 3 serogroups.

**Table 2:** Demographic and clinical characteristics of study participants, northern Tanzania, 2012–14.

		Acute leptospirosis (N = 24)		*Leptospira* seropositive (N = 252)		Controls (N = 592)	
		n	(%)	n	(%)	n	(%)
**Demographic characteristics**							
	Age, median (range) years	37.8 (2.4–67.7)		33.5 (0.3–93.5)		21.9 (0.2–84.2)	
	Female sex	13	(54.2)	147	(58.3)	313	(52.9)
	Pastoralist tribe*	0	(0)	6	(2.4)	2	(0.3)
	Residence in urban district	11	(45.8)	112	(44.4)	288	(48.7)
**Clinical history**							
	Fever duration >7 days	14	(58.3)		NA	160	(27.0)
	Received prior antibiotics	9	(37.5)		NA	219	(37.0)
	Conjunctival suffusion	0	(0)		NA	13	(2.2)
	Hemoptysis	0	(0)		NA	0	(0)
	Jaundice	0	(0)		NA	9	(1.5)
	Neck stiffness	6	(25)		NA	56	(9.5)
**Discharge diagnoses**		N = 16		N = 174		N = 341	
	Leptospirosis (clinical)	0	(0)	0	(0)	0	(0)
	Malaria (clinical)	4	(25.0)	30	(17.2)	89	(26.1)
	Malaria (laboratory)	0	(0)	5	(2.8)	11	(1.9)

Abbreviations: NA = Not applicable

*Pastoralist tribe: Maasai, Barahaig.

### Association of risk factors

Bivariable logistic regression of individual risk factors are included in S2 Table. There was a strong association between behaviors involving a single livestock species. For example having cleaned cattle waste was associated with having fed cattle with an OR 324.1 (95% confidence intervals 96.6–1087.0). There was some association between behaviors involving different livestock species. For example having cleaned cattle waste was associated with having cleaned goat waste with an OR 28.8, 95% confidence interval 12.0–69.1. There was a small magnitude association between rodent contact variables and livestock related variables. For example owning cattle was not associated with seeing rodents frequently in the house, compound or fields, and had a low magnitude association with seeing rodents in the kitchen or food store (OR 1.5, 95 confidence intervals 1.1–2.1).

### Acute leptospirosis individual behaviors

Results for the logistic regression analysis of individual behaviors are shown in Table 3. On bivariable regression, variables associated with acute leptospirosis included working in rice fields (OR 14.6, 95% confidence intervals (CI) 2.9–59.5); cleaning up cattle waste (OR 4.3, CI 1.2–12.9); feeding cattle (OR 3.9, CI 1.3–10.3) and working as a farmer (OR 3.3, CI 1.3–8.2).

**Table 3:** Bivariable logistic regression of individual risk factors for acute leptospirosis among patients with febrile illness (N = 616), northern Tanzania, 2012–14.

Variable		Acute leptospirosis (N = 24)		Controls (N = 592)		Bivariable logistic regression	
		n	(%)	n	(%)	OR (95% CI)	p value
**Livestock exposure variables**							
Cleaned cattle waste		5	(20.8)	34	(5.7)	4.3 (1.2–12.9)	<0.01
Cleaned goat waste		3	(12.5)	30	(5.1)	2.7 (0.48–9.7)	0.11
Cleaned pig waste		1	(4.2)	17	(2.9)	1.5 (0.03–10.2)	0.71
Fed cattle		7	(29.2)	57	(9.6)	3.9 (1.3–10.3)	0.02
Fed goats		4	(16.7)	57	(9.6)	1.9 (0.45–5.9)	0.26
Fed pigs		1	(4.2)	20	(3.8)	1.2 (0.02–8.5)	0.84
Herded cattle		1	(4.2)	6	(1.0)	4.2 (0.49–36.7)	0.19
Herded goats		1	(4.2)	13	(2.2)	1.9 (0.24–15.4)	0.53
Kept cattle inside the house		1	(4.2)	3	(0.5)	8.5 (0.15–110)	0.29
Kept goats inside the house		1	(4.2)	5	(0.8)	5.1 (0.10–48.2)	0.14
Kept pigs inside the house		1	(4.2)	54	(9.1)	0.4 (0.01–2.7)	0.42
Milked cattle		2	(8.3)	15	(2.5)	3.5 (0.36–16.5)	0.28
Milked goats		0	(0.0)	1	(0.2)	NA	
Owning cattle		9	(37.5)	128	(21.6)	2.2 (0.82–5.4)	0.12
Own dogs		3	(12.5)	110	(18.6)	0.6 (0.12–2.2)	0.66
Owned goats		8	(33.3)	143	(24.2)	1.6 (0.6–4.0)	0.31
Own pigs		1	(4.2)	54	(9.1)	0.43 (0.1–3.3)	0.70
Slaughtered cattle		3	(12.5)	51	(8.6)	1.5 (0.44–5.3)	0.51
Slaughtered goats		2	(8.3)	14	(2.4)	3.8 (0.80–17.5)	0.09
Slaughtered pigs		0	(0.0)	4	(0.7)	NA	
**Rodent exposure variables**							
Worked as a farmer		10	(41.7)	106	(17.9)	3.3 (1.3–8.2)	0.01
Killed rodents		3	(12.5)	15	(2.5)	5.5 (0.94–21.5)	0.06
Freq. rodents seen in house*							
	Less than once/week	2	(8.3)	96	(16.2)	0.74 (0.15–3.5)	0.70
	More than once/week	14	(58.3)	213	(36.0)	2.3 (0.96–5.6)	0.06
Freq. evidence of rodents seen in house*							
	Less than once/week	3	(12.5)	88	(14.9)	0.99 (0.27–3.7)	0.99
	More than once/week	11	(45.8)	214	(36.2)	1.5 (0.62–3.6)	0.37
Freq. rodents seen in fields*							
	Less than once/week	2	(8.3)	31	(5.2)	3.0 (0.83–10.9)	0.09
	More than once/week	5	(20.8)	63	(10.6)	2.5 (0.88–7.0)	0.09
Freq. rodents seen in compound*							
	Less than once/week	3	(12.5)	99	(16.7)	0.84 (0.23–3.0)	0.79
	More than once/week	9	(37.5)	161	(27.2)	1.5 (0.64–3.7)	0.33
Freq. rodents seen in kitchen/ food store*							
	Less than once/week	5	(20.8)	82	(13.9)	1.6 (0.56–4.6)	0.38
	More than once/week	5	(20.8)	142	(24.0)	0.9 (0.33–2.6)	0.88
**Surface water exposure variables**							
Bathed in surface water		6	(25.0)	124	(20.9)	1.3 (0.40–3.4)	0.79
Drank untreated surface water		4	(16.7)	69	(11.7)	1.2 (0.28–3.6)	0.97
Had standing water in compound		9	(37.5)	154	(26.1)	1.7 (0.64–4.2)	0.31
Walked barefoot		14	(58.3)	271	(45.8)	0.85 (0.33–2.1)	0.85
Washed in surface water		5	(20.8)	132	(22.3)	0.91 (0.26–2.6)	1.00
Worked in rice fields		4	(16.7)	8	(1.4)	14.6 (2.9–59.5)	<0.01

Abbreviations: OR = Odds ratio; CI = Confidence intervals; Freq = frequency

Key

* Reference category is ‘no rodents/ evidence of rodents seen in month prior’

### Acute leptospirosis exposure scales

**Fig 2:**
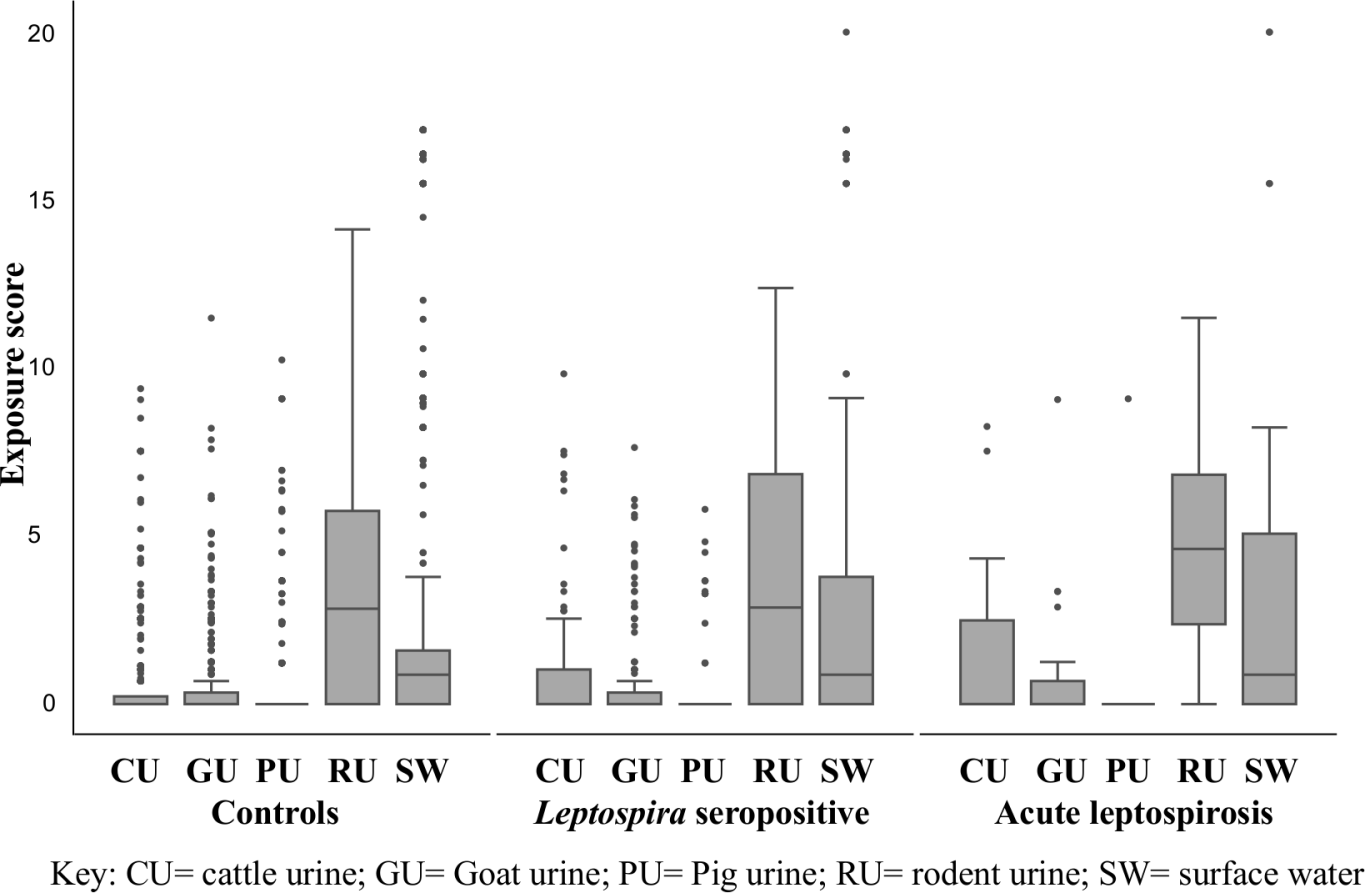
Participant scores of exposure to animal urine and surface water, northern Tanzania, 2012–14 (N = 844).

Nine (42.9%) of 21 experts (three livestock field officers, four veterinarians, and two zoonotic disease epidemiologists provided internally consistent multiple pairwise rankings of the relative exposure to livestock urine from the behaviors listed in Table 4. Four (100.0%) of four experts (one water engineer, one water and sanitation epidemiologist, and two zoonotic disease epidemiologists) provided consistent multiple pairwise rankings of the relative exposure to surface water. Three (75.0%) of four experts (one rodent ecologist, one veterinarian, and one zoonotic disease epidemiologist) provided consistent multiple pairwise rankings of the relative exposure to rodent urine. The individual behaviors evaluated for each exposure scale and the geometric means of the weights assigned to each are listed in Table 4. The results of pairwise comparisons, and calculated weights for each behavior are presented in S3 Table, S4 Table, and S5 Table. The distributions of participants’ exposure scores on each scale are shown in Fig 2. Overall, 534 (69.3%) of participants had no evidence of exposure to cattle urine, 563 (73.0%) had no exposure to goat urine, 241 (31.2%) had no exposure to rodent urine, and 262 (34.0%) had no exposure to surface water. There was limited correlation between cattle urine exposure and both goat urine exposure (r^2^ = 0.21) and pig urine exposure (r^2^ = 0.04). In addition there was little correlation between livestock urine exposure scores and rodent urine exposure (for example, cattle urine exposure and rodent urine exposure, r^2^ = 0.04), livestock exposure scores and surface water exposure (for example cattle urine and surface water (r^2^ = 0.02), and between rodent urine exposure and surface water exposure (r^2^ = 0.02). All exposure scales had a linear relationship with log odds of acute leptospirosis

**Table 4:** Component risk factors and relative weights for exposure to multiple leptospirosis infection sources derived from an analytic hierarchy process conducted among East African subject matter experts, 2015.

Cattle or goat urine exposure		Rodent urine exposure		Surface water exposure	
Variable	Weight	Variable	Weight	Variable	Weight
Clean livestock waste	0.85	Subsistence farmer	0.70	Drink surface water	1.81
Birth livestock	0.78	Sugar cane worker	0.68	Bathe in surface water	1.41
Keep livestock inside house	0.74	Handle rat carcasses	0.66	Work in rice field	0.73
Milk livestock	0.72	See rats in kitchen	0.55	Wash in surface water	0.65
Slaughter livestock	0.66	Plumber	0.54	Walk barefoot	0.22
Veterinarian	0.48	See rats in house	0.47	Have standing water in compound	0.18
Herd livestock	0.27	Kill rats	0.45		
Keep livestock around house	0.26	See evidence of rats in house	0.40		
Feed livestock	0.23	See evidence of rats in compound	0.28		
		See evidence of rats in fields	0.28		
Total	5.00		5.00		5.00

Our bivariable logistic regression (Table 5) found that increasing exposure to cattle urine (OR 2.3, CI 1.1–4.7) and exposure to rodents (OR 1.7, CI 1.1–2.8) were both associated with increased odds of acute leptospirosis. In multivariable logistic regression (Table 5), no exposure scale was independently associated with leptospirosis. As shown in S6 Table, there were no significant interactions. The largest variance inflation factor was 1.33.

**Table 5:** Bivariable and multivariable logistic regression models of association between exposure scales and acute leptospirosis among patients with febrile illness in northern Tanzania, 2012–14.

	Bivariable		Multivariable	
Variable	OR (95% CI)	p-value	OR (95% CI)	p-value
Cattle urine exposure	2.3 (1.1–4.7)	0.02	1.9 (0.91–4.0)	0.09
Goat urine exposure	2.0 (0.82–4.7)	0.13		
Pig urine exposure	1.0 (0.32–3.3)	0.97		
Rodent urine exposure	1.7 (1.1–2.8)	0.02	1.6 (0.98–2.6)	0.06
Surface water exposure	1.1 (0.84–1.4)	0.48		

Abbreviations: OR = odds ratio; CI = confidence interval

### Acute leptospirosis geospatial and temporal analysis

GPS co-ordinates were available for houses of 649 (84.2%) participants. No two or more participants lived at the same household. Land use designation could be determined from participant’s self-reported village of residence for an additional 79 (10.2%) participants. There was no evidence of clustering in the spatial distribution of cases. Results of the bivariable logistic regression analysis of geo-referenced variables and rainfall, and acute leptospirosis are shown in Table 6. There were no statistically significant associations.

**Table 6:** Bivariable logistic regression of temporal and geo-referenced risk factors for acute leptospirosis among patients with febrile illness, northern Tanzania, 2012–14.

Variable	Acute Leptospirosis (N = 17)		Controls (N = 504)		Acute leptospirosis logistic regression	
	n	(%)	n	(%)	OR (95% CI)	p-value
**Land use**						
Cultivated	12	(70.6)	330	(65.5)	REF	
Urban	4	(23.5)	148	(29.4)	0.74 (0.24–2.3)	0.61
Natural	1	(5.9)	26	(5.2)	1.1 (0.13–8.5)	0.96
**Main soil type**						
Chromic Luvisol	17	(100)	444	(88.1)	REF	
Other	0	(0)	60	(11.9)	NA	NA
**Ward population density, median in people/ km**^**2**^ **(IQR)**	2052 (433–7296)		962 (131–6064)		1.1* (0.95–1.2)	0.32
**Elevation, median in MASL (IQR)**	803 (794–856)		840 (803–980)		0.97§ (0.96–1.0)	0.25
**Annual Mean Rainfall**						
<1000mm	2	(11.8)	103	(20.4)	REF	
1000–1600mm	13	(76.5)	264	(52.4)	2.5 (0.56–11.4)	0.23
>1600mm	2	(11.8)	137	(27.2)	2.1 (0.44–9.7)	0.36
**Village zone designation**						
Urban	10	(58.8)	281	(55.8)	REF	
Peri-urban	3	(17.7)	104	(20.6)	0.81 (0.22–3.0)	0.75
Rural	4	(23.5)	119	(23.6)	0.94 (0.29–3.1)	0.92
**Temporal Rainfall Variables**	**N = 24**		**N = 592**			
Total rainfall in preceding 30 days, median in mm (IQR)	25 (1–65)		22 (1–68)		1.0 # (0.67–1.8)	0.69
Largest single day rainfall in preceding 30 days, median in mm (IQR)	13 (1–27)		13 (1–34)		0.92 # (0.16–5.2)	0.93

Abbreviations: IQR = interquartile range; MASL = meters above sea level; NA = not applicable; REF = reference value

Key

*The odds ratio, is per 100 people/ km^2^

§ Odds ratio is per 10m increase in elevation

♯ OR is per 100mm increase in rainfall

### *Leptospira* seropositivity

Results of the logistic regression of individual risk factors for *Leptospira* seropositivity are listed in Table 7. Working in rice fields (OR 3.6, 95% CI 1.5–9.0); slaughtering goats (OR 2.3, 95% CI 1.0–4.8), working as a farmer (OR 1.8, 95% CI 1.3–2.5), and frequently seeing rodents in the kitchen (OR 1.5, 95% CI 1.1–2.1) were significant risk factors (p < 0.05) on bivariable regression. We fitted an initial multivariable model using the risk factors shown in Table 8. As shown in S6 Table, we did not identify any significant interactions between variables. In our final multivariable model, working as a farmer (OR 1.6, CI 1.1–2.3), working in the rice fields (OR 2.7 CI 1.0–7.2), or seeing rodents in the kitchen ≥ once per week (OR 1.5, CI 1.0–2.1) were all independent risk factors for *Leptospira* seropositivity. Walking barefoot (OR 0.7, CI 0.5–0.9) and owning dogs (OR 0.6, CI 0.4–1.0) were associated with reduced odds of *Leptospira* seropositivity.

**Table 7:** Bivariable logistic regression of risk factors for *Leptospira* seropositivity among patients with febrie illness in northern Tanzania, 2012–14.

Variable		*Leptospira* seropositive (N = 252)		Controls (N = 592)		Bivariable logistic regression	
		n	(%)	n	(%)	OR (95% CI)	P value
**Livestock exposure variables**							
Cleaned cattle waste		18	(7.1)	34	(5.7)	1.3 (0.70–2.3)	0.44
Cleaned goat waste		17	(6.8)	30	(5.1)	1.4 (0.73–25)	0.33
Cleaned pig waste		7	(2.8)	17	(2.9)	1.0 (0.40–2.4)	0.94
Fed cattle		30	(11.9)	57	(9.6)	1.3 (0.79–2.0)	0.32
Fed goats		27	(10.7)	57	(9.6)	1.1(0.69–1.8)	0.63
Fed pigs		6	(2.4)	20	(3.4)	0.7 (0.28–1.8)	0.45
Herded cattle		4	(1.6)	6	(1.0)	1.5 (0.44–5.6)	0.48
Herded goats		10	(4.0)	13	(2.2)	1.8 (0.80–4.3)	0.15
Kept cattle inside the house		4	(1.6)	3	(0.5)	3.2 (0.70–14.3)	0.13
Kept goats inside the house		3	(1.2)	5	(0.8)	1.4 (0.34–6.0)	0.64
Kept pigs inside the house		0	(0.0)	0	(0.0)	NA	
Milked cattle		10	(4.0)	15	(2.5)	1.6 (0.70–3.6)	0.27
Milked goats		2	(0.8)	1	(0.2)	4.7 (0.43–52.4)	0.21
Owning cattle		64	(25.4)	128	(21.6)	1.2 (0.87–1.7)	0.23
Owned dogs		35	(13.9)	110	(18.6)	0.71 (0.47–1.1)	0.10
Owned goats		72	(28.6)	143	(24.2)	1.3 (0.90–1.8)	0.18
Owned pigs		15	(5.9)	54	(9.1)	0.63 (0.35–1.1)	0.13
Slaughtered cattle		22	(8.7)	51	(8.6)	1.0 (0.60–1.7)	0.96
Slaughtered goats		13	(5.2)	14	(2.4)	2.3 (1.0–4.8)	0.04
Slaughtered pigs		4	(1.6)	4	(0.7)	2.4 (0.59–9.6)	0.23
**Rodent exposure variables**							
Worked as a farmer		70	(27.9)	106	(17.9)	1.8 (1.3–2.5)	<0.01
Killed at least one rodent		6	(2.4)	15	(2.5)	0.9 (0.36–2.4)	0.89
Handled rodent carcasses		10	(4.0)	18	(3.0)	1.3 (0.60–2.9)	0.49
Freq. rodents seen in house*							
	Less than once/week	36	(14.3)	96	(16.2)	0.88 (0.57–1.4)	0.58
	More than once/week	96	(38.1)	213	(36.0)	1.1 (0.77–1.5)	0.71
Freq. rodents seen in kitchen*							
	Less than once/week	30	(11.9)	82	(13.9)	1.0 (0.6–1.5)	0.84
	More than once/week	81	(32.1)	142	(24.0)	1.5 (1.1–2.1)	0.02
Freq. rodents seen in compound*							
	Less than once/week	42	(16.7)	99	(16.7)	1.0 (0.68–1.6)	0.86
	More than once/week	74	(29.4)	161	(27.2)	1.1 (0.80–1.6)	0.51
Freq. rodents seen in fields*							
	Less than once/week	16	(6.4)	31	(5.2)	1.3 (0.67–2.3)	0.48
	More than once/week	31	(12.3)	63	(10.6)	1.2 (0.75–1.9)	0.45
**Surface water exposure variables**							
Bathed in surface water		56	(22.2)	124	(20.9)	1.1 (0.75–1.5)	0.68
Drank untreated surface water		41	(16.3)	69	(11.7)	1.1 (0.75–1.7)	0.56
Had standing water in compound		81	(32.1)	154	(26.1)	1.3 (0.97–1.9)	0.07
Walked barefoot		98	(38.9)	271	(45.8)	0.8 (0.56–1.0)	0.07
Washed in surface water		60	(23.8)	132	(22.3)	1.1 (0.77–1.5)	0.63
Worked in rice fields		12	(4.8)	8	(1.4)	3.6 (1.5–9.0)	0.01

Abbreviations: OR = odds ratio; CI = confidence interval; Freq. = Frequency

Key

* Reference category is ‘no rodents/ evidence of rodents seen in month prior’

**Table 8:** Multivariable logistic regression of individual risk factors *Leptospira* seropositivity among patients with febrile illness in northern Tanzania, 2012–14.

Variable	Multivariate logistic regression	
	OR (95% CI)	P value
Herded goats		
Kept cattle inside		
Owned dogs	0.63 (0.41–0.99)	0.05
Owned goats	1.4 (0.96–2.0)	0.08
Owned pigs	0.64 (0.34–1.2)	0.16
Slaughtered goats		
Worked as a farmer	1.6 (1.1–2.3)	0.02
Had standing water in compound	1.4 (0.99–1.9)	0.06
Walked barefoot	0.67 (0.49–0.92)	0.01
Worked in rice fields	2.7 (1.0–7.2)	0.05
Saw rodents in the kitchen ≤ once per week*	0.89 (0.55–1.4)	0.63
Saw rodents in the kitchen > once per week*	1.4 (1.0–2.1)	0.03

Footnote: Variables included in the table were included in the initial model. Those with an OR were included in the final model. Abbreviations: OR = odds ratio; CI = confidence interval.

Key

* Reference category is ‘no rodents/ evidence of rodents seen in month prior’

The logistic regression models of the exposure scales and *Leptospira* seropositivity are shown in Table 9. Increasing exposure to rodent urine (OR1.2, CI 1.0–1.5) was associated with *Leptospira* seropositivity on bivariable logistic regression, but not on multivariable regression.

**Table 9:** Bivariable and multivariable logistic regression models of association of exposure scales and *Leptospira* seropositivity among patients with febrile illness in northern Tanzania, 2012–14.

	Bivariable		Multivariable	
Variable	OR (95% CI)	p-value	OR (95% CI)	p-value
Cattle urine exposure	1.2 (0.86–1.8)	0.23		
Goat urine exposure	1.5 (0.96–1.3)	0.07	1.3 (0.86–2.1)	0.20
Pig urine exposure	0.89 (0.56–1.4)	0.61		
Rodent urine exposure	1.2 (1.0–1.1.5)	0.03	1.2 (0.98–1.4)	0.07
Surface water exposure	1.0 (0.93–1.1)	0.47		

Abbreviations: OR = odds ratio; CI = confidence interval

Results of the bivariable logistic regression analysis of rainfall and *Leptospira* seropositivity are shown in Table 10. There was an inverse association with mean annual rainfall >1,600mm per year (OR 0.56, 95% CI 0.33–0.93). We fitted an initial multivariable model using household elevation, mean annual rainfall, maximum daily rainfall in the preceding 30 days, and total rainfall in the preceding 30 days. The final model contained elevation (OR 0.99 per 10m, CI 0.98–1.0, p = 0.06), and total rainfall in the preceding 30 days (OR 1.2 per 100mm, CI 0.95–1.5, p = 0.13) but neither association was statistically significant. An analysis of the risk factors for seropositivity against *Leptospira* serogroup Icterohaemorrhagiae is included as S6 Table.

**Table 10:** Bivaraite logistic regression of temporal and geo-referenced risk factors for *Leptospira* seropositivity among patients with febrile illness, in northern Tanzania, 2012–14.

Variable	*Leptospira* seropositivity (N = 181)		Controls (N = 504)		*Leptospira* seropositivity logistic regression	
	n	(%)	n	(%)	OR (95% CI)	p-value
**Land use**						
Cultivated	122	(67.4)	330	(65.5)	REF	
Urban	51	(28.2)	148	(29.4)	0.93 (0.64–1.4)	0.72
Natural	8	(4.4)	26	(5.2)	0.83 (0.37–1.9)	0.66
**Main soil type**						
Chromic Luvisol	162	(89.5)	444	(88.1)	REF	
Other	19	(11.5)	60	(11.9)	0.87 (0.50–1.5)	0.61
**Ward population density, median in people/ km**^**2**^ **(IQR)**	1172 (310–7296)		962 (131–6064)		1.0 *(0.98–1.1)	0.26
**Elevation, median in MASL (IQR)**	822 (796–945)		840 (803–980)		0.99§ (0.98–1.0)	0.05
**Annual Mean Rainfall**						
<1000mm	46	(25.4)	103	(20.4)	REF	
1000–1600mm	101	(55.8)	264	(52.4)	0.86 (0.56–1.3)	0.47
>1600mm	34	(18.8)	137	(27.2)	0.56 (0.33–0.93)	0.02
**Village zone designation**						
Urban	92	(50.8)	281	(55.8)	REF	
Peri-urban	39	(21.6)	104	(20.6)	1.5 (0.74–1.8)	0.54
Rural	50	(27.6)	119	(23.6)	1.3 (0.86–1.9)	0.23
**Temporal Rainfall Variables**	**N = 252**		**N = 592**			
Total rainfall in preceding 30 days, median in mm (IQR)	33 (2–72)		22 (1–68)		1.2♯ (1.0–1.5)	0.06
Largest single day rainfall in preceding 30 days,	14 (1–35)		13 (1–34)		1.6♯ (0.88–2.9)	0.12

Abbreviations: OR = Odds ratio IQR = interquartile range; MASL = Meters above sea level

Key

*OR is per 1000 person/km^2^ increase in density

§ OR is per 10m increase in elevation

♯ OR is per 100mm increase in rainfall

## Discussion

We identified multiple associations between exposure to cattle and acute leptospirosis, suggesting that cattle may be important sources of human leptospirosis in northern Tanzania. We also identified work in rice fields as an important risk factor for human leptospirosis. These findings must be interpreted with caution, as they were based on a small number of cases, and were present in only bivariable regression. Despite this, our findings have implications for the control and prevention of leptospirosis in Tanzania.

On bivariable regression, exposure to cattle was associated with acute human leptospirosis both when we evaluated individual behaviors and scales of cumulative exposure to cattle urine. These findings support other data from northern Tanzania that indicate that livestock may be an important source of human leptospirosis [31]. Among cattle slaughtered for meat in the Moshi area, 7.6% of cattle tested were carrying pathogenic *Leptospira* spp. in their kidneys [31]. Furthermore, seroreactivity against serogroups Australis and Sejroe, the two dominant serogroups among human cases in our study, was also frequently observed among cattle slaughtered for meat in the Moshi area in 2014 [12]. Our findings are also consistent with studies examining risk factors for *Leptospira* seropositivity in Africa. *Leptospira* seropositivity was common among abattoir workers in Kenya and Tanzania [11, 27]. In rural Uganda, livestock skinning was reported as a risk factor for seroreactivity and human seropositivity to livestock-associated *Leptospira* serovars was common [28]. In a global context, cattle have also been identified as a key risk factor in other rural livestock-farming communities in Central America and South Asia [14, 15], suggesting that strategies to reduce either livestock leptospirosis or transmission of leptospirosis from livestock to humans may be important global public health interventions.

Rodent exposure is an important risk factor for leptospirosis in the tropics, particularly in urban areas of Asia and South America [4, 29, 30]. In our study, an increasing score on the exposure to rodent urine scale was associated with acute leptospirosis in bivariable regression. However, the only individual component of the scale for which we found an association on bivariable regression was smallholder farming. Since smallholder farming may involve substantial exposure to both livestock and rodents, and other rodent related variables were not associated with leptospirosis the role of rodents in this association is uncertain. We also found that frequently sighting rodents in the kitchen or food store was associated with *Leptospira* seropositivity. Rodents could transmit leptospirosis to humans, or act as a reservoir that transmit *Leptospira* to livestock. However, recent work in the Kilimanjaro Region found no evidence of *Leptospira* urinary shedding, or renal infection among 393 wild rodents [31]

Although practiced by few participants, we found an association between working in rice fields, and both acute leptospirosis and *Leptospira* seropositivity. In some areas of northern Tanzania rice farming is practiced intensively, and there are active efforts to increase irrigated, continuously flooded rice farming across Tanzania [32]. In Asia rice farming is an established risk factor for leptospirosis. In Asia humans are infected through prolonged contact with water that may be contaminated by infected animal hosts [4, 29]. Further work is needed to evaluate possible sources of contamination of rice paddies in Tanzania and promote personal protective measures among rice farmers.

We did not find associations between acute leptospirosis and rainfall, or environmental risk factors around the home. The small number of cases available for analysis, and the relative lack of resolution of geo-referenced data meant that this result must be interpreted with caution. The lack of association with heavy rainfall differs from findings of studies from other locations [33, 34]. We found that seropositivity was associated with lower elevation and lower rainfall. While we did not have household level slope data, the topography of the study area includes steeply sloping terrain on the flanks of Mount Kilimanjaro that may not favor surface water accumulation. The lack of association between leptospirosis and home location may indicate that the workplace is an important site for infection [9, 11]. Future studies should collect data regarding workplace location.

Clinicians did not diagnose leptospirosis during the study period, and over-diagnosis of malaria was common. At the time of our study, there were no locally available, accurate diagnostic tests for leptospirosis. In addition, despite the high incidence in the region, clinician awareness of leptospirosis and other zoonotic diseases remains low [35]. This highlights the need for clinician education and evaluations in Africa of inexpensive point-of-care diagnostic tests.

We found that risk factors and the pattern of predominant reactive serogroups among leptospirosis cases was markedly different from those in seropositive individuals, for whom the febrile illness concurrent with enrollment was unlikely to be leptospirosis. In particular, reactivity to serogroup Icterohaemorrhagiae was common among seropositive participants, but there were few acute cases associated with this serogroup. These results may indicate that a serovar from the Icterohaemorrhagiae serogroup was circulating in this region [36], causing only mild disease not requiring tertiary medical care. Elsewhere, a difference in severity of disease has been linked to variability of infecting *Leptospira* species [37], Alternatively, the presence of Icterohaemorrhagiae seropositivity but absence of acute cases could indicate historic circulation of this serogroup that has since declined. Other results suggest that leptospirosis has a dynamic epidemiology in this area with the emergence and decline of specific serovars over time [3]. Cross reactivity between serogroups, and non-specific reactivity are other possible explanations [38].

Our study had several limitations. First, the prevalence of acute leptospirosis was lower than anticipated [8], potentially curtailing our ability to detect important associations. Conversely, associations of individual activities and leptospirosis identified by this study were sometimes based on only a few cases and should be interpreted with caution, especially given the multiple statistical tests. In addition, changes in leptospirosis incidence in the study area might also reflect changes in predominant sources and modes of transmission over time [3]. Second, the associations for acute leptospirosis were seen only on bivariable analysis, and these associations may be due to confounding from unobserved behaviors. Due to the complex interconnection between individual behaviours, we also consider that confounding may influence the multivariable logistic regression model of individual behaviours and *Leptospira* seropositivity. For example, the inverse association of walking barefoot and leptospirosis is puzzling, and we think it is likely to be influenced by an association with some protective factor, despite not identifying such an association among the behaviors we investigated. Diagnostic test limitations may have also introduced classification errors of participant cases or controls into our analysis. Leptospirosis is notoriously difficult to diagnose, particularly in the acute stages of illness and all currently available diagnostic tests for leptospirosis, including MAT [39], are imperfect. The sensitivity of MAT on paired serum samples is approximately 80% and the specificity close to 100% [40]. Specifically, not all participants with leptospirosis will seroconvert [40], and it is not possible to differentiate between historic and recent infection based on a single high titer [41]. We chose MAT for our case definitions since MAT on paired serum samples, while imperfect, remains the reference standard [40]. Furthermore, culture, nucleic acid amplification and point-of-care IgM serology lack sensitivity in our setting [12, 42, 43], and reports from other settings have been mixed [39, 44–46]. Our MAT panel comprising 20 serovars covered the major *Leptospira* serogroups that cause human disease, and all those within which African isolates are grouped [1]. We did not use locally isolated serovars and this may have influenced identification of cases. However, studies on the use of local isolates in MAT reference panels have shown that they do not necessarily perform better than other serovars from the same serogroup [47, 48]. Our analysis of acute leptospirosis was limited to cases across all serogroups. We acknowledge that risk factors may vary by infecting serovar, and pan-serogroup analyses may mask important associations.

We developed scales for use in our analyses for dimension reduction due to the unanticipated low number of cases. We suggest that cumulative exposure scales may have a future role in assessing sources of acute leptospirosis, as they allow assessment of cumulative exposure that may be important in assessing individual risk of disease. The analytic hierarchy process was an appropriate method of creating these scales, as it is an effective tool for quantifying multi-dimensional qualitative knowledge [24]. While we acknowledge that there is scope to improve our cumulative exposure scales, our scales that quantify expert opinion offer more biologically plausible groupings than statistical methods of dimension reduction. Key areas for future development of cumulative exposure scales are to validate them across multiple groups of experts, and to formally compare their effectiveness against purely statistical dimension reduction. Since our questionnaire sought exposures over a 30 day period, recall bias may have influenced our findings. Finally, we enrolled only 47.1% of eligible patients. We found no bias towards particular ethnic or occupational groups. However, we cannot rule out the possibility that the enrollment pattern influenced our results. Despite these limitations, the consistency of the association of the livestock related variables strengthens our confidence in the interpretation of their role in transmitting leptospirosis to people in our region.

Our results have implications for control of leptospirosis. Transmission of leptospirosis within rice fields, and from livestock to people is amenable to control through personal protective equipment for those performing high risk activities [49]. In addition, *Leptospira* vaccines are available for use in livestock against some *Leptospira* serovars. In some countries such vaccines have contributed to successful control of leptospirosis [49]. However, before a vaccination program is considered it is essential to understand reservoir structure and predominant infecting serovars.

Our study identifies associations between cattle contact and work in rice fields with acute leptospirosis. Our findings suggest that cattle may be a source of human leptospirosis in northern Tanzania. Further work is needed to determine if these findings are stable over time, and to investigate the link by isolating infecting serovars from humans and animal hosts. The development of local MAT capacity, or use of nucleic acid amplification or point-of-care IgM tests that have sufficiently high sensitivity would enable real-time diagnosis and allow testing of potential animal hosts living in proximity to humans with acute leptospirosis. Nonetheless, our findings suggest that control of *Leptospira* infection in livestock could play a role in preventing human leptospirosis in Africa.

## Supporting information

S1 Text Participant questionnaire assessing risk factors for zoonotic disease among patients with febrile illness in northern Tanzania, 2012–14. (PDF)

S1 Table Spearman’s correlation coefficients for individual behavior variables among patients with febrile illness in northern Tanzania, 2012–14. (XLSX)

S2 Table Matrix of pairwise comparisons made by experts in East African livestock urine exposure during analytic hierarchy process, and calculation of livestock urine exposure weights, 2015. (XLSX)

S3 Table Matrix of pairwise comparisons made by experts in rodent urine exposure in East Africa during analytic hierarchy process, and calculation of rodent urine exposure weights, 2015. (XLSX)

S4 Table Matrix of pairwise comparisons made by experts in surface water exposure in East Africa during analytic hierarchy process, and calculation of surface water exposure weights, 2015. (XLSX)

S5 Table Interaction terms assessed during logistic regression analyses of risk factors for acute leptospirosis and *Leptospira* seropositivity in northern Tanzania, 2012–14. (DOCX)

S6 Table Results tables for logistic regression of risk factors for seropositivity to *Leptospira* serogroup Icterohaemorrhagiae among patients with febrile illness in northern Tanzania. (DOCX)
