# Supplementary material for: Risk factors for human acute leptospirosis in northern Tanzania
Source: PLoS Negl Trop Dis. 2018 Jun 7;12(6):e0006372. doi: 10.1371/journal.pntd.0006372 (PMC5991637; doi:10.1371/journal.pntd.0006372)
Supplement: S1 Text — (PDF) [file pntd.0006372.s001.pdf]

5308616076

BACTERIAL ZOONOSES STUDY QUESTIONNAIRE

Patient Identification number (PID)

129 - ☐☐☐☐ - ☐

Date of admission (dd/mm/yyyy)

☐☐ / ☐☐ / ☐☐☐☐

☐☐☐ Interviewer's initials

Study Identification number (SID)

FEVER - ☐☐☐☐ - ☐

Date of birth (dd/mm/yyyy)

☐☐ / ☐☐ / ☐☐☐☐

Sex

○ Male ○ Female

Primary informant

○ Patient
○ Parent/Guardian/Legal Rep
○ Relative
○ Other

Location:

○ MRH - M ○ KCMC M1
○ MRH - F ○ KCMC M2
○ MRH Peds ○ KCMC M2 Private
○ MRH OPD ○ KCMC ICU

SECTION 1

1.1 Which region do you live in?

○ Kilimanjaro ○ Dodoma
○ Arusha ○ Manyara
○ Tanga ○ Other
○ Singida

1.2 *If Kilimanjaro region*, what district do you live in?

○ Moshi rural ○ Mwanga
○ Moshi urban ○ Same
○ Hai ○ Siha
○ Rombo

1.3 What village do you live in?

☐☐☐☐☐☐☐☐☐☐☐☐☐☐☐☐☐

1.4 Village code

☐☐☐☐☐☐☐

1.5 What is your tribe?

○ Chagga ○ Pare ○ Maasai ○ Sambaa ○ Other

☐☐☐☐☐☐☐☐☐☐☐☐☐☐☐☐☐

1.6 In the past month, have you traveled outside your home region?

○ Yes ○ No

1.7 *If yes,* to which region(s) have you traveled and for how long?

○ Kilimanjaro ○ Dodoma
○ Arusha ○ Manyara
○ Tanga ○ Other
○ Singida ☐☐☐☐☐☐☐☐☐☐

☐☐ Days (total days outside of home region, max 31 days)

1.8 How many years of education have you had?

○ No education
○ Primary (1-7 years)
○ Seconday (8-11 years)
○ High school (12-13 years)
○ University/college

1.9 What is your marital status?

○ Married
○ Single
○ Divorced/separated
○ Widowed

1.10 How many adults live in your household? (age 18 years or older)

☐☐

1.11 How many children live in your household? (age less than 18 years)

☐☐

1.12 How long have you lived in your current home?

☐☐ Years

OR if less than 1 year

☐☐ Months

OR if less than 1 month

☐☐ Days

☐☐☐☐☐

SECTION 2: Water Use and Exposure

2.1 In the past month, where did you get: /***Katika kipindi cha mwezi uliopita,*** *ni wapi ulipata:*

Drinking water/*Maji ya kunywa*

	**Primary source (choose only one)** *Chanzo cha kudumu (chagua moja tu)*	**Secondary source (choose only one)** *Chanzo mbadala (chagua moja tu)*	**Other sources (Choose all that apply)** *Vianzo vingine (chagua yote yanayohusika)*
**Piped water into the home 1** *Maji ya bomba nyumbani*	○	○	○
**Public/communal well or standpipe 2** *Kisima cha umma, bomba ya umma*	○	○	○
**River or creek (moving water) directly 3** *Moja kwa moja kutoka katika mto au mfereji (maji yanayotembea)*	○	○	○
**Lake, pond, dam (standing water) directly 4** *Moja kwa moja kutoka katika ziwa, dimbwi, au bwawa (maji yaliyosimama)*	○	○	○
**Private well or pump 5** *Kisima au pampu binafsi*	○	○	○
**From a spring 6** *Moja kwa moja kutoka katika chemchem*	○	○	○
**Rainwater 7** *Maji ya mvua*	○	○	○
**Tanker truck 8** *Gari la kubebea maji (boza)*	○	○	○
**Cart or wheelbarrow with small tank or drum 9** *Toroli/mkokoteni wa kubebea maji*	○	○	○
**Bottled water 10** *Maji ya chupa*	○	○	○
**Other (specify) 11** *Vinginevyo (ainisha)*	○	○	○

2.2 In the past month, where did you get: / ***Katika kipindi cha mwezi uliopita,*** *ni wapi ulipata:*

Bathing water/*Maji ya kuoga*

	**Primary source (choose only one)** *Chanzo cha kudumu (chagua moja tu)*	**Secondary source (choose only one)** *Chanzo mbadala (chagua moja tu)*	**Other sources (Choose all that apply)** *Vianzo vingine (chagua yote yanayohusika)*
**Piped water into the home 1** *Maji ya bomba nyumbani*	○	○	○
**Public/communal well or standpipe 2** *Kisima cha umma, bomba ya umma*	○	○	○
**River or creek (moving water) directly 3** *Moja kwa moja kutoka katika mto au mfereji (maji yanayotembea)*	○	○	○
**Lake, pond, dam (standing water) directly 4** *Moja kwa moja kutoka katika ziwa, dimbwi, au bwawa (maji yaliyosimama)*	○	○	○
**Private well or pump 5** *Kisima au pampu binafsi*	○	○	○
**From a spring 6** *Moja kwa moja kutoka katika chemchem*	○	○	○
**Rainwater 7** *Maji ya mvua*	○	○	○
**Tanker truck 8** *Gari la kubebea maji (boza)*	○	○	○
**Cart or wheelbarrow with small tank or drum 9** *Toroli/mkokoteni wa kubebea maji*	○	○	○
**Other (specify) 11** *Vinginevyo, ainisha*	○	○	○

2.3 In the past month, where did you get:/***Katika kipindi cha mwezi uliopita,*** *ni wapi ulipata:*

Washing water (clothes, dishes)/ *Maji ya matumizi ya kuosha (kufua, kuosha vyombo)*

	**Primary source (choose only one)** *Chanzo cha kudumu (chagua moja tu)*	**Secondary source (choose only one)** *Chanzo mbadala (chagua moja tu)*	**Other sources (Choose all that apply)** *Vianzo vingine (chagua yote yanayohusika)*
**Piped water into the home 1** *Maji ya bomba nyumbani*	O	O	O
**Public/communal well or standpipe 2** *Kisima cha umma, bomba ya umma*	O	O	O
**River or creek (moving water) directly 3** *Moja kwa moja kutoka katika mto au mfereji (maji yanayotembea)*	O	O	O
**Lake, pond, dam (standing water) directly 4** *Moja kwa moja kutoka katika ziwa, dimbwi, au bwawa (maji yaliyosimama)*	O	O	O
**Private well or pump 5** *Kisima au pampu ya binafsi*	O	O	O
**From a spring 6** *Moja kwa moja kutoka katika chemchem*	O	O	O
**Rainwater 7** *Maji ya mvua*	O	O	O
**Tanker truck 8** *Gari la kubebea maji (boza)*	O	O	O
**Cart or wheelbarrow with small tank or drum 9** *Toroli/mkokoteni wa kubebea maji*	O	O	O
**Other (specify) 11** *Vinginevyo, ainisha*	O ☐	O ☐	O ☐

2.4 Is your drinking water treated (by filtering, boiling, chlorinating, straining, etc.)?
Huwa una tibu maji ya kunywa (kwa kuchuja, kuchemsha, kuweka dawa ya klorine, kuchuja kwa nguo, nk)?

O Yes O No

2.5 *If yes*, how do you treat it? (choose all that apply)
Kama ndio, unatibu vipi ? (chagua yote yanayohusika)

- O Boiling/ kuchemsha
- O Strain it through a cloth/ kuchuja kwa nguo
- O Adding disinfectant, such as chlorine or bleach/ kuweka dawa klorine/bleach/water guard
- O Sedimentation and decant/ kuacha kwa muda yatwae/uchafu uende chini
- O Filtering/ kuchuja
- O Solar disinfection/ kuweka juani
- O Other, specify/ nyinginezo, ainisha ☐☐☐☐☐☐☐☐☐☐☐☐☐☐☐

2.6 Did you go into water (river, creek, lake, dam, pond) in the past month to bathe, swim, wash clothes, collect water, fish, take animals for water, or for any other reason?
Je wewe uliingia katika maji (mto, mfereji, ziwa, bwawa, dimbwi) katika kipindi cha mwezi uliopita kuoga, kuogelea, kufua, kuchota maji, kuvua, kunywesha mifugo, au kwa sababu nyingine yoyote?

O Yes O No

If yes, how many days per week (1-7)?
Kama ndio, *siku ngapi kwa juma (1-7)?* siku ☐

2.7 Have you walked outside barefoot in the past month?
Je wewe ulitembea bila kuvaa viatu (peku) nje katika kipindi cha mwezi moja uliopita?

○ Yes ○ No

If yes, how many days per week (1-7)? ***Kama ndio,*** *siku ngapi kwa juma (1-7)?* siku ☐

How many of these days was it muddy? *Siku ngapi ya hizo kulikuwa na tope?* siku ☐

2.8 Have you walked outside in sandals with open toes or flip flops the past month?
Je wewe ulitembea nje ukiwa umevaa viatu vya wazi (kandabili) na vidole vya miguu kuwa wazi katika kipindi cha mwezi uliopita?

○ Yes ○ No

If yes, how many days per week (1-7)? ***Kama ndio,*** *siku ngapi kwa juma (1-7)?* siku ☐

How many of these days was it muddy? *Siku ngapi ya hizo kulikuwa na tope?* siku ☐

2.9 In the past month, has there been standing water in your compound following rains?
Katika kipindi cha mwezi uliopita, kumewahi kuwa na maji yaliyotuama katika uwanja wako au nyumba yako baada ya mvua kunyesha?

○ Yes ○ No

2.10 Have you had cuts, scratches or scrapes on your hands/arms or feet/legs in the past month?
Je wewe umekuwa na sehemu uliyokatwa, mkwaruzo au mchubuko katika mkono/kiganja au miguu katika kipindi cha mwezi uliopita?

○ Yes ○ No

SECTION 3: Home and Land

3.1 Was mud or manure added to your house in the past month?
Kulikuwa na tope au mbolea (ya wanyama) iliyokandikwa katika nyumba yako katika kipindi cha mwezi uliopita?

○ Yes ○ No

3.2 ***If yes***, did you add the mud or manure yourself?
Kama ndio, je ni wewe uliweka matope au mbolea mwenyewe?

○ Yes ○ No

3.3 What is the roof of your house made of? (choose only one)
Je paa la nyumba yako limeezekwa na nini? (chagua moja tu)

○ Metal/bati

○ Thatch/nyasi

○ Wood/mbao

○ Tiles/vigae

○ Cement/sementi

○ Other/nyinginezo ainisha ☐☐☐☐☐☐☐☐☐☐☐☐☐☐☐

3.5 What is the floor of your house made of? (choose all that apply)
Sakafu ya nyumba yako imetengenezwa na nini? (chagua yote yanayohusika)

○ Dirt or mud/ udongo au tope

○ Dung/ kinyesi cha mifugo

○ Brick/ matofali

○ Cement/ sementi

○ Tile or linoleum/ vigae au sakafu ya mpira

○ Wood or planks/ mbao

○ Other/ nyinginezo ainisha ☐☐☐☐☐☐☐☐☐☐☐☐☐☐

3.4 What are the walls of your house made of? (choose all that apply)
Ukuta wa nyumba yako umetengenezwa na nini? (chagua yote yanayohusika)

○ Mud or manure/tope au kinyesi cha mifugo

○ Burnt brick/matofari yaliyochomwa

○ Mud bricks (uncooked)/matofali ya udongo

○ Cement/sementi

○ Wood or planks/mbao au fito

○ stone/ mawe

○ thatch/ nyasi

○ Other/nyinginezo ainisha ☐☐☐☐☐☐☐☐☐☐☐☐☐☐☐

☐☐☐☐☐

3.6 What type of toilet system do members of your household use? (choose only one)
Ni aina gani ya mfumo wa choo ambao unatumika na wakazi wa kaya yako? (chagua moja tu)

- ○ **Flush or pour toilet with septic tank, including squat toilet**
  Choo cha kuchuchumaa, cha kumwaga maji na mfumo wa shimo la maji taka
- ○ **Flush or pour toilet connected to sewer pipe**
  Choo cha maji kilichounganishwa na bomba la maji taka
- ○ **Pit latrine with covering slab**
  Choo cha shimo kilichosakafiwa
- ○ **Pit latrine without covering slab**
  Choo cha shimo ambacho hakijasakafiwa (kisichosakafiwa)
- ○ **Ventilated improved pit latrine (VIP)**
  Choo cha shimo bora chenye bomba la kutoa hewa chafu (VIP)
- ○ **Bucket or plastic bags**
  Ndoo au mfuko
- ○ **No facilites or field or bush**
  Hakuna choo, kwenda porini

3.7 Do you have any of the following items in your household? (choose all that apply)
Kuna chochote kati ya hivi katika nyumba yako? (chagua yote yanayohusika)

- ○ Mobile phone/simu ya mkononi
- ○ Radio/redio
- ○ Television/luninga
- ○ Sofa/makochi
- ○ Bicycle/baiskeli
- ○ Motorbike/pikipiki
- ○ Car/gari
- ○ none of these/ sina chochote kati ya hivi

3.8 How many rooms are in your house?
Kuna vyumba vingapi katika nyumba yako?

☐☐

SECTION 4: Animals

4.1 Does anyone in your household own any of these animals?
Kuna yeyote katika kaya yako anamiliki yeyote kati ya hawa wanyama?

		Number of animals *Wanyama wangapi*	Location/ *Eneo* (choose all that apply): Inside your house *Ndani ya nyumba yako*	Around your house *Nje ya nyumba yako*	Distant location *Mbali na nyumba yako (hauwezi kuwaona kila mwezi)*
cattle/ng'ombe	○ Yes ○ No	☐☐☐	○	○	○
goats/mbuzi	○ Yes ○ No	☐☐☐	○	○	○
pigs/nguruwe	○ Yes ○ No	☐☐☐	○	○	○
chickens/kuku	○ Yes ○ No	☐☐☐	○	○	○
sheep/kondoo	○ Yes ○ No	☐☐☐	○	○	○
dogs/mbwa	○ Yes ○ No	☐☐☐	○	○	○
donkeys/punda	○ Yes ○ No	☐☐☐	○	○	○
horses/farasi	○ Yes ○ No	☐☐☐	○	○	○
cats/paka	○ Yes ○ No	☐☐☐	○	○	○
ducks/bata	○ Yes ○ No	☐☐☐	○	○	○
rabbits/sungura	○ Yes ○ No	☐☐☐	○	○	○
other/wengine specify	○ Yes ○ No				
☐☐☐☐☐☐☐☐☐☐		☐☐☐	○	○	○
☐☐☐☐☐☐☐☐☐☐		☐☐☐	○	○	○

4.2 Have you fed any animals in the past month? If yes, how many days in a typical week?
Je wewe umelisha yeyote kati ya wanyama hao katika kipindi cha mwezi mmoja uliopita? Kama ndio, siku ngapi katika wiki ya kawaida?

○ Yes ○ No

		Number of days (1-7)
cattle/ng'ombe	○ Yes ○ No	☐
goats/mbuzi	○ Yes ○ No	☐
pigs/nguruwe	○ Yes ○ No	☐
chickens/kuku	○ Yes ○ No	☐
sheep/kondoo	○ Yes ○ No	☐
dogs/mbwa	○ Yes ○ No	☐
donkeys/punda	○ Yes ○ No	☐
horses/farasi	○ Yes ○ No	☐
cats/paka	○ Yes ○ No	☐
ducks/bata	○ Yes ○ No	☐
rabbits/sungura	○ Yes ○ No	☐
other/wengine specify	○ Yes ○ No	
☐☐☐☐☐☐☐☐☐☐		☐
☐☐☐☐☐☐☐☐☐☐		☐

4.3 Have you milked any of these animals in the past month? If yes, how many days in a typical week?
Je wewe umemkamua yeyote kati ya wanyama hawa katika kipindi cha mwezi uliopita? Kama ndio, siku ngapi katika wiki (katika wiki ya kawaida)?

O Yes O No

		Number of days (1-7)
cattle/ng'ombe	O Yes O No	☐
goats/mbuzi	O Yes O No	☐
sheep/kondoo	O Yes O No	☐
other/wengineo specify ☐☐☐☐☐☐☐☐☐☐☐	O Yes O No	☐

4.5 Have you herded or used any of these animals for herding in the past month? If yes, how many days in a typical week?
Je wewe umechunga au tumia yeyote kati ya wanyama hawa kwa ajili ya kuchungia katika kipindi cha mwezi uliopita? Kama ndio, siku ngapi katika wiki (katika wiki ya kawaida)?

O Yes O No

		Number of days (1-7)
cattle/ng'ombe	O Yes O No	☐
goats/mbuzi	O Yes O No	☐
sheep/kondoo	O Yes O No	☐
dogs/mbwa	O Yes O No	☐
other/wengineo specify ☐☐☐☐☐☐☐☐☐☐☐	O Yes O No	☐

4.4 Have you removed/cleaned up the waste of any of these animals in the past month? If yes, how many days in a typical week?
Je wewe umetoa/safisha uchafu/mbolea kutoka kwa mnyama yoyote kati ya hawa katika kipindi cha mwezi uliopita? Kama nidio, siku ngapi katika wiki (katika wiki ya kawaida)?

O Yes O No

		Number of days (1-7)
cattle/ng'ombe	O Yes O No	☐
goats/mbuzi	O Yes O No	☐
pigs/nguruwe	O Yes O No	☐
chickens/kuku	O Yes O No	☐
sheep/kondoo	O Yes O No	☐
dogs/mbwa	O Yes O No	☐
donkeys/punda	O Yes O No	☐
horses/farasi	O Yes O No	☐
cats/paka	O Yes O No	☐
ducks/bata	O Yes O No	☐
rabbits/sungura	O Yes O No	☐
other/wengineo specify ☐☐☐☐☐☐☐☐☐☐☐ ☐☐☐☐☐☐☐☐☐☐☐	O Yes O No	☐ ☐

4.6 Have you assisted with the birthing of any animals in the past month?
Je wewe ulisaidia katika kuzalisha mnyama yeyote katika mifugo katika kipindi cha mwezi uliopita?

O Yes O No

4.7 If yes, which animals did you assist with birthing?
Kama ndio, ni mnyama gani uliyemsaidia kuzalisha?

cattle/ng'ombe	O Yes O No
goats/mbuzi	O Yes O No
pigs/nguruwe	O Yes O No
sheep/kondoo	O Yes O No
dogs/mbwa	O Yes O No
donkeys/punda	O Yes O No
horses/farasi	O Yes O No
cats/paka	O Yes O No
other/wengineo specify ☐☐☐☐☐☐☐☐☐☐☐☐	O Yes O No

6522616075

4.8 Have any of your family's animals died in the past month? (do not include animals intentionally slaughtered)
Kuna yeyote kati ya mifugo ya familia yako amekufa katika kipindi cha mwezi uliopita?

○ Yes ○ No

4.9 *If yes*, which animals and how many?
Kama ndio, wanyama gani na wangapi?

		Number adult *wanyama wakubwa wangapi*	Number young *wanyama watoto wangapi*
cattle/ng'ombe	○ Yes ○ No	☐☐	☐☐
goats/mbuzi	○ Yes ○ No	☐☐	☐☐
pigs/nguruwe	○ Yes ○ No	☐☐	☐☐
chickens/kuku	○ Yes ○ No	☐☐	☐☐
sheep/kondoo	○ Yes ○ No	☐☐	☐☐
dogs/mbwa	○ Yes ○ No	☐☐	☐☐
donkeys/punda	○ Yes ○ No	☐☐	☐☐
horses/farasi	○ Yes ○ No	☐☐	☐☐
cats/paka	○ Yes ○ No	☐☐	☐☐
ducks/bata	○ Yes ○ No	☐☐	☐☐
rabbits/sungura	○ Yes ○ No	☐☐	☐☐
other/wengineo specify	○ Yes ○ No		
☐☐☐☐☐☐☐☐☐☐☐		☐☐	☐☐
☐☐☐☐☐☐☐☐☐☐☐		☐☐	☐☐

4.10 Has milk production changed in any of your cattle in the past 3 months?
Je uzalishaji wa maziwa ulibadilika katika ng'ombe yoyote katika kipindi miezi mitatu iliopita?

○ Yes ○ No

4.11 If yes, has milk production increased or decreased?
Kama ndio, je uzalishaji wa maziwa umeongezeka au umepungua?

○ Increased/ kuongezeka ○ Decreased/ kupungua

4.12 Have any of your animals had joint problems in the past 3 months?
Je kuna yeyote kati ya wanyama wako amekuwa na matatizo ya viungo katika kipindi miezi 3 iliopita?

○ Yes ○ No

4.13 *If yes,* which animals? *Iwapo ndio, mnyama gani?*

cattle/ng'ombe	○ Yes	○ No
goats/mbuzi	○ Yes	○ No
sheep/kondoo	○ Yes	○ No

4.14 Have any of your family's animals aborted or had stillbirths in the past month?
Je kuna yeyote kati ya wanyama wa kwenye familia yako mimba imetoka au kuzaa mtoto mfu katika kipindi cha mwezi uliopita?

○ Yes ○ No

4.15 *If yes*, which animals had stillbirths or abortions and how many?
Kama ndio, mnyama gani mimba imetoka au amezaa mtoto mfu na wangapi katika mwezi uliopita?

cattle/ng'ombe	○ Yes ○ No	☐☐
goats/mbuzi	○ Yes ○ No	☐☐
pigs/nguruwe	○ Yes ○ No	☐☐
sheep/kondoo	○ Yes ○ No	☐☐
dogs/mbwa	○ Yes ○ No	☐☐
donkeys/punda	○ Yes ○ No	☐☐
horses/farasi	○ Yes ○ No	☐☐
cats/paka	○ Yes ○ No	☐☐
rabbits/sungura	○ Yes ○ No	☐☐
other/wengineo ☐☐☐☐☐☐☐☐☐☐☐☐	○ Yes ○ No	☐☐

4.16 *If yes*, did you handle any of the aborted birth products?
Kama ndio, je wewe ulishika chochote kati ya sehemu za mimba iliotoka au vilivyozaliwa?

○ Yes ○ No

4.17 Have you slaughtered or butchered (or assisted in slaughtering or butchering) any livestock or domesticated animals in the past month?
Je wewe ulichinja au kukatakata (au kusaidia kuchinja au kukatakata) yeyote kati ya mifugo au wanyama wanaofugwa katika kipindi cha mwezi uliopita?

○ Yes ○ No

cattle/ng'ombe	○ Yes ○ No
goats/mbuzi	○ Yes ○ No
pigs/nguruwe	○ Yes ○ No
sheep/kondoo	○ Yes ○ No
chickens/kuku	○ Yes ○ No
ducks/bata	○ Yes ○ No
rabbits/sungura	○ Yes ○ No
other/wengineo specify	○ Yes ○ No
☐☐☐☐☐☐☐☐☐☐☐☐☐	

4.18 Did you kill (or assist in killing) any wild rodents in the past month?
Je wewe uliua(au kusaidia kumwua) yeyote kati ya panya pori katika kipindi cha mwezi uliopita?

○ Yes ○ No

4.19 Did you kill (or assist in killing) any wild animals in the past month? *If yes,* which animals?
Je wewe uliua (au kusaidia kuua) yeyote kati ya wanyama wa porini katika kipindi cha mwezi uliopita?

○ Yes ○ No

monkey/ape family (eg monkeys chimpanzees, baboons, apes) *jamii ya nyani/sokwemtu (mfano, kima, nyani, sokwemtu)*	○ Yes ○ No
buffalo/*nyati*	○ Yes ○ No
antelope/*swala*	○ Yes ○ No
hippo/*kiboko*	○ Yes ○ No
other/wengineo specify ☐☐☐☐☐☐☐☐☐☐☐☐	○ Yes ○ No

4.20 Have you handled carcasses of any animals in the past month? *If yes*, of which animals?
Je wewe umeshika mzoga wa mnyama yeyote katika kipindi cha mwezi uliopita?
○ Yes ○ No

cattle/*ng'ombe*	○ Yes ○ No
goats/*mbuzi*	○ Yes ○ No
pigs/*nguruwe*	○ Yes ○ No
chickens/*kuku*	○ Yes ○ No
sheep/*kondoo*	○ Yes ○ No
dogs/*mbwa*	○ Yes ○ No
donkeys/*punda*	○ Yes ○ No
cats/*paka*	○ Yes ○ No
ducks/*bata*	○ Yes ○ No
hedgehogs/*karunguyeye*	○ Yes ○ No
rabbits/*sungura*	○ Yes ○ No
wild rodents/*panya pori*	○ Yes ○ No
monkey/ape family (monkeys chimpanzees, baboons, apes) *jamii ya nyani/sokwemtu (mfano, kima, nyani, sokwemtu)*	○ Yes ○ No
buffalo/*nyati*	○ Yes ○ No
antelope/*swala*	○ Yes ○ No
hippo/*kiboko*	○ Yes ○ No
other/*wengineo* specify ☐☐☐☐☐☐☐☐☐☐☐☐ ☐☐☐☐☐☐☐☐☐☐☐☐	○ Yes ○ No

4.21 Other than animals owned by your household, have you seen any of these animals around your village or neighborhood in the past month? How many days in a typical week?
Tofauti na wanyama ambao wanamilikiwa na wakazi wa kaya yako, umewahi kuona yeyote kati ya wanyama hawa katika kijiji au eneo lako katika kipindi cha mwezi uliopita? Siku ngapi katika wiki ya kawaida, umewaona wanyama hao ?

		Number of days (1-7)
cattle/ng'ombe	○ Yes ○ No	☐
goats/mbuzi	○ Yes ○ No	☐
pigs/nguruwe	○ Yes ○ No	☐
chickens/kuku	○ Yes ○ No	☐
sheep/kondoo	○ Yes ○ No	☐
dogs/mbwa	○ Yes ○ No	☐
donkeys/punda	○ Yes ○ No	☐
horses/farasi	○ Yes ○ No	☐
cats/paka	○ Yes ○ No	☐
ducks/bata	○ Yes ○ No	☐
rats/panya wakubwa	○ Yes ○ No	☐
mice/panya wadogo	○ Yes ○ No	☐
field rats/panya buku	○ Yes ○ No	☐
hedgehogs/karunguyeye	○ Yes ○ No	☐
monkeys/kima	○ Yes ○ No	☐
baboons/sokwemtu	○ Yes ○ No	☐
elephants/tembo	○ Yes ○ No	☐
zebra/pundamilia	○ Yes ○ No	☐
buffalo/nyati	○ Yes ○ No	☐
antelope/swala	○ Yes ○ No	☐
hippos/viboko	○ Yes ○ No	☐
jackel/mbweha	○ Yes ○ No	☐
mongoose/kicheche	○ Yes ○ No	☐
bats/popo	○ Yes ○ No	☐
other/wengineo ☐☐☐☐☐☐☐☐☐☐☐☐ ☐☐☐☐☐☐☐☐☐☐☐☐	○ Yes ○ No	☐ ☐

2773616079

SECTION 5: Food, Rodent and Agricultural Exposures

5.1 In the past month, how many days a week have you consumed the following types of dairy products?
Katika kipindi cha mwezi uliopita, ni siku ngapi katika wiki ambazo umetumia aina za mazao ya maziwa zifuatazo?

		Number of days (1-7)
pasteurized or boiled milk<br>*maziwa ya kiwandani au yaliyochemshwa*	O Yes O No	☐
raw milk<br>*maziwa mabichi*	O Yes O No	☐
cheese made from raw milk<br>*jibini za maziwa mabichi*	O Yes O No	☐
butter made from raw milk<br>*siagi iliyotengenezwa na maziwa mabichi*	O Yes O No	☐
cream made from raw milk<br>*mafuta ya maziwa mabichi*	O Yes O No	☐
yogurt made from raw milk<br>*mgando/mtindi ya maziwa mabichi*	O Yes O No	☐
other products made from raw milk<br>*mazao mengine kutoka katika maziwa mabichi* ☐☐☐☐☐☐☐☐	O Yes O No	☐
cheese, butter, cream or yogurt from boiled or pasteurized milk<br>*Jibini, siagi, mafuta au mgando/mtindi kutoka katika maziwa yaliyochemshwa au ya kiwandani*	O Yes O No	☐
cheese, butter, cream or yogurt but unsure whether milk raw or boiled/pasteurized<br>*Jibini, siagi, mafuta au mgando/mtindi lakini hakuna uhakika kama maziwa yalikuwa mabichi, yaliyochemshwa au ya kiwandani*	O Yes O No	☐

5.2 If raw milk or products from raw milk were consumed, where did they come from? (Leave blank If no raw milk consumed in past month)
Iwapo maziwa yalikuwa mabichi au mazao kutoka maziwa mabichi yalitumika, yalitoka wapi?

shop/*duka*	O Yes O No
open market/ *sokoni*	O Yes O No
neighbor/friend/family/<br>*jirani/rafiki/familia*	O Yes O No
from own cow/*ng'ombe wako*	O Yes O No
from own goats/*mbuzi wako*	O Yes O No
from own sheep/*kondoo wako*	O Yes O No

5.3 In the past month, have you eaten rodents?
Katika kipindi cha mwezi uliopita, umekula panya buku?

O Yes O No

5.4 *If yes*, how many days per week?
Kama ndio, siku ngapi katika juma?
siku ☐

5.5 In the past month, have you consumed any of the following types of raw meat or offal or raw animal blood? How many days per week?
Katika kipindi cha mwezi uliopita, je wewe umewahi kutumia chechote kati ya aina ya nyama mbichi au nyama za ndani, au damu mbichi ya mnyama? Siku ngapi katika juma?

		Number of days (1-7)
raw cow blood<br>*damu mbichi ya ng'ombe (ikiwa ni pamoja na iliyochanganywa na supu, kisusio)*	O Yes O No	☐
raw goat blood<br>*damu mbichi ya mbuzi (ikiwa ni pamoja na iliyochanganywa na supu, kisusio)*	O Yes O No	☐
raw sheep blood<br>*damu mbichi ya kondoo (ikiwa ni pamoja na iliyochanganywa na supu, kisusio)*	O Yes O No	☐
raw blood from other animal<br>*damu mbichi ya mnyama mwingine* ☐☐☐☐☐☐☐☐☐☐	O Yes O No	☐
raw meat or offal from cow<br>*nyama mbichi au za ndani kutoka kwa ng'ombe*	O Yes O No	☐
raw meat or offal from goat<br>*nyama mbichi au za ndani kutoka ya mbuzi*	O Yes O No	☐
raw meat or offal from sheep<br>*nyama mbichi au za ndani kutoka kwa* kuku	O Yes O No	☐
raw meat or offal from chicken<br>*nyama mbichi au za ndani kutoka kwa* kuku	O Yes O No	☐
raw meat or offal from pig<br>*nyama mbichi au za ndani kutoka kwa nguruwe*	O Yes O No	☐
raw meat or offal from another animal<br>*nyama mbichi au za ndani kutoka kwa mnyama mwingineo* ☐☐☐☐☐☐☐☐☐☐	O Yes O No	☐

5.6 Have you done agricultural work in the fields or farm in the past month? This includes tending crops or harvesting fruits (including bananas), vegetables. grains or coffee.
Je umewahi kufanya kazi za kilimo au za shamba katika kipindi cha mwezi uliopita? Hii inahusisha kuhudumia mazao au kuvuna matunda (pamoja na ndizi), mboga, nafaka au kahawa.

O Yes O No

5.7 Have you worked in the sugarcane fields in the past month?
Je umewahi kufanya kazi katika shamba la miwa katika kipindi cha mwezi uliopita?

O Yes O No

5.8 Have you worked in the garden in the past month?
Je umewahi kufanya kazi katika bustani katika kipindi cha mwezi uliopita?

O Yes O No

5.9 Have you worked in the rice fields in the past month?
Je umewahi kufanya kazi katika shamba la mpunga katika kipindi cha mwezi uliopita?

○ Yes ○ No

5.10 When doing agricultural work have you used or worn any of the following items in the past month? (Leave blank if no agricultural work done)
Wakati wa kufanya kazi za kilimo, umewahi kutumia au kuvaa chochote kati ya vitu vifuatavyo katika kipindi cha mwezi uliopita?

boots or closed shoes<br>*mabuti au viatu vya kufunika miguu*	○ Yes ○ No
open shoes (sandals or flip flops)<br>*viatu vya wazi (ndala au kandambili)*	○ Yes ○ No
gloves<br>*glovu*	○ Yes ○ No
barefeet<br>*miguu wazi/peku*	○ Yes ○ No

5.11 Have you seen rodents in your house in the last month?
Je umewahi kuwaona panya katika nyumba yako katika kipindi cha mwezi uliopita?

○ less than once a week *siyo zaidi ya mara moja kwa juma* ○ more than once a week *zaidi ya mara moja kwa juma* ○ never *kamwe sijaona*

5.12 Have you seen evidence of rodents (such as feces, urine, rodent tracks, heard noises) in your house in the last month?
Je umewahi kuona ushahidi wa panya (kama choo, mkojo, sauti, njia yake) katika nyumba yako katika kipindi cha mwezi uliopita?

○ less than once a week ○ more than once a week ○ never

5.13 Have you seen rodents or evidence of rodents in your kitchen or stored food in the last month?
Je umewahi kuwaona panya au ushahidi wakuwepo katika jiko lako au chakula kilichohifadhiwa katika kipindi cha mwezi uliopita?

○ less than once a week ○ more than once a week ○ never

5.14 Have you seen rodents or evidence of rodents in your compound in the last month?
Je umewahi kuwaona panya au ushahidi wakuwepo panya katika eneo la nyumba yako lako katika kipindi cha mwezi uliopita?

○ less than once a week ○ more than once a week ○ never

5.15 Have you seen rodents or evidence of rodents in your fields in the last month?
Je umewahi kuwaona panya au ushahidi wakuwepo panya katika shamba lako katika kipindi cha mwezi uliopita?

○ less than once a week ○ more than once a week ○ never
○ dont have fields

SECTION 6: Occupation

6.1 What are your main professional activities?
Ajira yako kuu ni ipi? (ainisha zote zinazohusika, onyesha kazi ya msingi)

	Primary occupation (choose one)<br>*Kazi ya kudumu*	Other occupation (choose all)<br>*Kazi ya ziada*
rancher/livestock attendant 1<br>*mchungi/mfugaji*	○	○
farmer/field or agricultural worker 2<br>*mkulima/mfanya kazi wa shambani*	○	○
gardener 3<br>*mtunza bustani*	○	○
miner 4<br>*mchimba madini*	○	○
military 5<br>*mwanajeshi*	○	○
butcher/abbatoir worker 6<br>*mchinjaji/mfanyakazi wa machinjioni*	○	○
wildlife worker 7<br>*mfanyakazi wa wanyamapori*	○	○
milk supplier 8<br>*msambazaji wa maziwa*	○	○
outdoor manual labor 9<br>*kibarua wa kazi za nje*	○	○
artisan/craftsman 10<br>*mhunzi*	○	○
veterinarian 11<br>*mganga wa mifugo*	○	○
housewife 12<br>*mama wa nyumbani*	○	○
office worker or student 13<br>*mfanyakazi wa ofisini au mwanafunzi*	○	○
healthcare worker 14<br>*mfanyakazi wa afya*	○	○
merchant/trader 15<br>*mjasiriamali/mfanyabiashara*	○	○
teacher 16<br>*mwalimu*	○	○
driver 17<br>*dereva*	○	○
sewer worker 18<br>*wazibua vyoo*	○	○
guard/police 19<br>*askari/polisi*	○	○
unemployed 20<br>*sijaajiriwa*	○	
other 21<br>nyingineo	○	
