# Supplementary material for: Risk factors for human acute leptospirosis in northern Tanzania
Source: PLoS Negl Trop Dis. 2018 Jun 7;12(6):e0006372. doi: 10.1371/journal.pntd.0006372 (PMC5991637; doi:10.1371/journal.pntd.0006372)
Supplement: S5 Table — (DOCX) [file pntd.0006372.s006.docx]

S5 Table. Interaction terms assessed during logistic regression analyses of risk factors for acute leptospirosis and *Leptospira* seropositivity in northern Tanzania, 2012-14

**Table A. Logistic regression of interaction terms and acute leptospirosis among patients with febrile illness in northern Tanzania, 2012-14**

| **Interaction** | **Odds ratio** | **95% CI** | **P value** |
| --- | --- | --- | --- |
| Cattle urine exposure and goat urine exposure | 1.9 | (0.47-7.3) | 0.38 |
| Cattle urine exposure and pig urine exposure | NA | NA | NA |
| Cattle urine exposure and rodent urine exposure | 0.95 | (0.38-2.4) | 0.91 |
| Cattle urine exposure and surface water exposure | 0.91 | (0.57-1.5) | 0.69 |
| Goat urine exposure and pig urine exposure | NA |  | 0.99 |
| Goat urine exposure and rodent urine exposure | 0.42 | (0.11-1.6) | 0.20 |
| Goat urine exposure and surface water exposure | 1.1 | (0.56-2.1) | 0.83 |
| Pig urine exposure and rodent urine exposure | 1.4 | (0.24-7.8) | 0.73 |
| Pig urine exposure and surface water exposure | NA | NA | NA |
| Rodent urine exposure and surface water exposure | 0.74 | (0.54-1.0) | 0.07 |

Abbreviations: OR = Odds ratio; CI= Confidence interval; NA = Not applicable: unable to be assessed

**Table B. Logistic regression of interaction terms and *Leptospira* seropositivity among patients with febrile illness in northern Tanzania, 2012-14**

| **Interaction** | **OR (95% CI)** | **P value** |
| --- | --- | --- |
| Standing water in the compound and owning cattle | 0.94 (0.43-2.1) | 0.89 |
| Standing water in the compound and owning goats | 1.3 (0.62-2.8) | 0.47 |
| Standing water in the compound and owning pigs | 1.4 (0.35-5.6) | 0.64 |
| Standing water in the compound and walking barefoot | 0.76 (0.40-1.5) | 0.41 |
| Walking barefoot and owning cattle | 1.1 (0.54-2.2) | 0.82 |
| Walking barefoot and owning goats | 1.3 (0.65-2.5) | 0.49 |
| Walking barefoot and owning pigs | 2.4 (0.73-8.1) | 0.15 |
| Working in rice fields and owning cattle | 2.2 (0.25-33.4) | 0.39 |
| Working in rice fields and owning goats | NA |  |
| Working in rice fields and owning pigs | NA |  |
| Working in rice fields and seeing rodents in the fields | 1.2 (0.40-3.6) | 0.74 |

Abbreviations: OR= odds ratio; CI= confidence interval

**Table C. Logistic regression of interaction terms and *Leptospira* seropositivity among patients with *Leptospira* seropositivity and controls in northern Tanzania, 2012-14**

| **Interaction** | **OR** | **(95% CI)** | **P value** |
| --- | --- | --- | --- |
| Cattle urine exposure and goat urine exposure | 1.1 | (0.60-2.1) | 0.70 |
| Cattle urine exposure and pig urine exposure | 1.3 | (0.63-2.7) | 0.48 |
| Cattle urine exposure and rodent urine exposure | 1.2 | (0.72-1.9) | 0.54 |
| Cattle urine exposure and surface water exposure | 1.1 | (0.85-1.4) | 0.56 |
| Goat urine exposure and pig urine exposure | 1.0 | (0.51-2.1) | 0.93 |
| Goat urine exposure and rodent urine exposure | 0.94 | (0.54-1.6) | 0.84 |
| Goat urine exposure and surface water exposure | 1.0 | (0.76-1.3) | 0.97 |
| Pig urine exposure and rodent urine exposure | 1.0 | (0.56-1.9) | 0.90 |
| Pig urine exposure and surface water exposure | 0.92 | (0.68-1.2) | 0.57 |
| Rodent urine exposure and surface water exposure | 0.97 | (0.86-1.1) | 0.61 |

Abbreviations: OR= odds ratio; CI= confidence interval
