# Supplementary material for: Risk factors for human acute leptospirosis in northern Tanzania
Source: PLoS Negl Trop Dis. 2018 Jun 7;12(6):e0006372. doi: 10.1371/journal.pntd.0006372 (PMC5991637; doi:10.1371/journal.pntd.0006372)
Supplement: S6 Table — (DOCX) [file pntd.0006372.s007.docx]

**S6 Table.**

**Table A. Logistic regression of risk factors for seropositivity with *Leptospira* serogroup Icterohaemorrhagiae among patients with febrile illness in northern Tanzania, 2012-14**

| **Variable** | ***Leptospira* serogroup Icterohaemorrhagiae seropositive (N=151)** | | **Controls (N=693)** | | **Bivariate logistic regression** | |
| --- | --- | --- | --- | --- | --- | --- |
|  | n | (%) | n | (%) | OR (95% CI) | P value |
| **Livestock exposure variables** |  |  |  |  |  |  |
| Cleaned cattle waste | 9 | (6.0) | 43 | (6.2) | 0.95 (0.45-2.0) | 0.91 |
| Cleaned goat waste | 7 | (4.6) | 40 | (5.8) | 0.79 (0.35-1.8) | 0.58 |
| Cleaned pig waste | 4 | (2.7) | 20 | (2.9) | 0.91 (0.32-2.7) | 0.87 |
| Fed cattle | 17 | (11.3) | 70 | (10.1) | 1.1 (0.64-2.0) | 0.67 |
| Fed goats | 15 | (9.9) | 69 | (10.0) | 1.0 (0.55-1.8) | 0.99 |
| Fed pigs | 2 | (1.3) | 24 | (3.5) | 0.37 (0.09-1.6) | 0.19 |
| Herded cattle | 2 | (1.3) | 8 | (1.2) | 1.1 (0.24-5.5) | 0.86 |
| Herded goats | 4 | (2.7) | 19 | (2.7) | 0.97 (0.32-2.9) | 0.95 |
| Kept cattle inside the house | 1 | (0.7) | 6 | (0.9) | 0.76 (0.09-6.4) | 0.80 |
| Kept goats inside the house | 0 | (0.0) | 8 | (1.2) | NA |  |
| Kept pigs inside the house | 8 | (5.3) | 60 | (8.7) | 0.59 (0.28-1.3) | 0.17 |
| Milked cattle | 3 | (2.0) | 22 | (3.2) | 0.62 (0.18-2.1) | 0.44 |
| Milked goats | 2 | (1.3) | 1 | (0.1) | 9.3 (0.84-103.1) | 0.07 |
| Owning cattle | 32 | (21.2) | 160 | (23.1) | 0.90 (0.58-1.4) | 0.62 |
| Owned dogs | 18 | (11.9) | 127 | (18.3) | 0.60 (0.36-1.0) | 0.06 |
| Owned goats | 41 | (27.2) | 174 | (25.1) | 1.1 (0.74-1.65) | 0.60 |
| Owned pigs | 9 | (6.0) | 142 | (8.7) | 0.67 (0.32-1.4) | 0.28 |
| Slaughtered cattle | 15 | (9.9) | 58 | (8.4) | 1.2 (0.66-2.2) | 0.54 |
| Slaughtered goats | 10 | (6.6) | 17 | (2.5) | 2.8 (1.3-6.3) | 0.01 |
| Slaughtered pigs | 3 | (2.0) | 5 | (0.7) | 2.7 (0.66-11.8) | 0.16 |
| **Rodent exposure variables** |  |  |  |  |  |  |
| Worked as a farmer | 143 | (20.6) | 33 | (22.0) | 1.1 (0.7-1.7) | 0.71 |
| Killed at least one rodent | 3 | (2.0) | 18 | (2.6) | 0.76 (0.22-2.6) | 0.66 |
| Handled rodent carcasses | 8 | (5.3) | 20 | (2.9) | 1.9 (0.81-4.4) | 0.14 |
| Freq. rodents seen in house  ≤once/week  >once/week | 16  58 | (10.6)  (38.4) | 116  251 | (16.7)  (36.2) | 0.58 (0.32-1.0)  0.98 (0.67-1.4) | 0.07  0.91 |
| Freq. rodents seen in kitchen  ≤once/week  >once/week | 12  55 | (8.0)  (36.4) | 100  168 | (14.4)  (24.4) | 0.61 (0.32-1.2)  1.7 (1.1-2.4) | 0.13  0.01 |
| Freq. rodents seen in compound  ≤once/week  >once/week | 21  44 | (13.9)  (29.1) | 120  191 | (17.3)  (27.6) | 0.78 (0.46-1.3)  1.0 (0.68-1.5) | 0.34  0.91 |
| Freq. rodents seen in fields  ≤once/week  >once/week | 7  19 | (4.6)  (12.6) | 40  75 | (5.8)  (10.8) | 0.81 (0.35-1.8)  1.2 (0.68-2.0) | 0.62  0.57 |
| **Surface water exposure variables** | | |  |  |  |  |
| Bathed in surface water | 34 | (22.5) | 146 | (21.1) | 1.1 (0.71-1.7) | 0.69 |
| Drank untreated surface water | 25 | (16.6) | 103 | (14.9) | 1.1 (0.71-1.8) | 0.60 |
| Had standing water in compound | 54 | (35.8) | 181 | (26.2) | 1.6 (1.1-2.3) | 0.02 |
| Walked barefoot | 39 | (25.8) | 153 | (22.1) | 0.91 (0.63-1.3) | 0.59 |
| Washed in surface water | 63 | (41.7) | 306 | (44.2) | 1.2 (0.82-1.8) | 0.32 |
| Worked in rice fields | 4 | (2.7) | 16 | (2.3) | 1.2 (0.38-3.5) | 0.80 |
| Abbreviations: OR= odds ratio; CI= confidence interval | | | | | | |

**Table B. Multivariate logistic regression of risk factors for seropositivity to *Leptospira* serogroup Icterohaemorrhagiae among patients with febrile illness in northern Tanzania, 2012-14**

| Variable | OR | 95% CI | P value |
| --- | --- | --- | --- |
| Fed pigs |  |  |  |
| Kept pigs inside |  |  |  |
| Owned dogs | 0.56 | (0.33-0.96) | 0.03 |
| Slaughtered goats | 2.9 | (1.2-6.6) | 0.01 |
| Slaughtered pigs |  |  |  |
| Seen rodents in the house ≤once per week | 0.58 | (0.31-1.1) | 0.10 |
| Seen rodents in the house >once per week | 0.50 | (0.29-0.89) | 0.01 |
| Seen rodents in the kitchen ≤once per week | 0.85 | (0.41-1.7) | 0.65 |
| Seen rodents in the kitchen >once per week | 2.6 | (1.5-4.6) | <0.01 |
| Had standing water in compound | 1.5 | (1.0-2.2) | 0.04 |

Abbreviations: OR= odds ratio; CI= confidence interval

**Table C. Bivariable and multivariable logistic regression models of association of exposure scales and seropositivity to *Leptospira* serogroup Icterohaemorrhagiae among patients with febrile illness in northern Tanzania, 2012-14**

|  | Bivariable* | |
| --- | --- | --- |
| Variable | OR (95% CI) | p-value |
| Cattle urine exposure | 0.96 (0.60-1.5) | 0.86 |
| Goat urine exposure | 1.2 (0.70-1.9) | 0.57 |
| Pig urine exposure | 0.83 (0.47-1.5) | 0.53 |
| Rodent urine exposure | 1.1 (0.91-1.4) | 0.29 |
| Surface water exposure | 1.0 (0.92-1.2) | 0.48 |
| Abbreviations: OR= odds ratio; CI= confidence interval | | |

*The multivariable results are not presented, as the best multivariable model did not fit better than individual components alone (AIC 797.3 vs 795.8)
